# Bayesian, Likelihood-Free Modelling of Phenotypic Plasticity and Variability in Individuals and Populations

**DOI:** 10.3389/fgene.2019.00727

**Published:** 2019-09-20

**Authors:** Joao A.N. Filipe, Ilias Kyriazakis

**Affiliations:** Agriculture, School of Natural and Environmental Sciences, Newcastle University, Newcastle upon Tyne, United Kingdom

**Keywords:** non-parametric, Bayesian inference, individual variation, phenotypic plasticity, reaction norms

## Abstract

There is a paradigm shift from the traditional focus on the “average” individual towards the definition and analysis of trait variation within individual life-history and among individuals in populations. This is a result of increasing availability of individual phenotypic data. The shift allows the use of genetic and environment-driven variations to assess robustness to challenge, gain greater understanding of organismal biological processes, or deliver individual-targeted treatments or genetic selection. These consequences apply, in particular, to variation in ontogenetic growth. We propose an approach to parameterise mathematical models of individual traits (e.g., reaction norms, growth curves) that address two challenges: 1) Estimation of individual traits while making minimal assumptions about data distribution and correlation, addressed *via* Approximate Bayesian Computation (a form of nonparametric inference). We are motivated by the fact that available information on distribution of biological data is often less precise than assumed by conventional likelihood functions. 2) Scaling-up to population phenotype distributions while facilitating unbiased use of individual data; this is addressed *via* a probabilistic framework where population distributions build on separately-inferred individual distributions and individual-trait interpretability is preserved. The approach is tested against Bayesian likelihood-based inference, by fitting weight and energy intake growth models to animal data and normal- and skewed-distributed simulated data. i) Individual inferences were accurate and robust to changes in data distribution and sample size; in particular, median-based predictions were more robust than maximum- likelihood-based curves. These results suggest that the approach gives reliable inferences using few observations and monitoring resources. ii) At the population level, each individual contributed *via* a specific data distribution, and population phenotype estimates were not disproportionally influenced by outlier individuals. Indices measuring population phenotype variation can be derived for study comparisons. The approach offers an alternative for estimating trait variability in biological systems that may be reliable for various applications, for example, in genetics, health, and individualised nutrition, while using fewer assumptions and fewer empirical observations. In livestock breeding, the potentially greater accuracy of trait estimation (without specification of multitrait variance-covariance parameters) could lead to improved selection and to more decisive estimates of trait heritability.

## Introduction

Phenotypic variation within and among biologically similar organisms (i.e., of the same species or same breed) drives population processes such as evolution (Roche et al., 2016), selective breeding (Hill, 2010), and disease epidemiology (Weiss et al., 2012). This variation can be used, for example, to target treatments or to study organism-level biological processes (Westneat et al., 2015). As technological and computational advances facilitate the measurement and analysis of individual traits, analytical and modelling paradigms in biological and medical research shift from a focus on the “average individual” to characterisations of individuals within populations and of populations using detailed individual traits. This shift is happening in diverse fields, such as health and physiology (Goaillard et al., 2009; Marder, 2011; Weiss et al., 2012; Britton et al., 2013; O’Leary and Marder, 2014; Mirams et al., 2016), ontogenetics and development (Speakman et al., 2004; Nilsson-Ortman et al., 2015; Beaman et al., 2016), ecology and evolution (Nussey et al., 2007; Forsman, 2015), behaviour (Dingemanse et al., 2010; Dingemanse and Wolf, 2013; Roche et al., 2016), and plant and animal agricultural production and food supply systems (Wolfert et al., 2017).

Trait variation among individuals or within an individual’s life history relates to genetic characteristics and to phenotypic plasticity, that is, an organism’s ability to undergo individual-specific trait changes in response to external stimuli (Nussey et al., 2007; Forsman, 2015; Roche et al., 2016). Mathematical models are a common tool to characterise phenotype expression. In ontogenesis, for example, models are used to summarise or predict responses to environmental or dietary change *via* reaction norms (Stearns and Koella, 1986; Nussey et al., 2007; Dingemanse et al., 2010) or to describe growth traits, such as size, body composition, and metabolism *via* nonlinear relationships (Emmans and Kyriazakis, 2001; Coyne et al., 2017; Filipe et al., 2018). In this paper, we introduce a statistical approach to fit models to individual observations and to estimate individual- and population-level trait distributions.

Statistical approaches for fitting population phenotype models to multiple-individual multiple-observation data, while capturing variation at within- and between-individual scales, have been under development and testing. For example, extensions of statistical regression frameworks, such as double hierarchical generalised linear models (DHGLM) (Lee and Nelder, 2006), have been proposed to allow for individual specificity (random effects) in both the mean and residual (deviation from mean) components of regression phenotype models. The mean usually incorporates relationships between traits and inherent factors, environment factors, age, and so on. The residuals model (known as dispersion or noise) has both population-wide and individual-specific parameters [often for a multivariate normal (MVN) distribution]. The DHGLM framework has been developed and applied in a variety of fields (San Cristobal-Gaudy et al., 1998; Hill and Mulder, 2010; Ronnegard et al., 2013; Cleasby et al., 2015), mostly as a frequentist but also as a Bayesian approach (Sorensen and Waagepetersen, 2003; Westneat et al., 2013). There are two important pillars to this framework. First, it is flexible in allowing alternative assumptions within conventional structures and can, for example, model long-dispersed (non-normal) residual distributions at the population level. However, the approach builds on multiple nested distributional assumptions that may or may not be valid in a given system and on the estimation of multiple mean and variance-covariance parameters to define them (Hill and Mulder, 2010; Cleasby et al., 2015). Second, this framework is necessarily a compromise in how data are deployed to inform individual-level parameters and population-wide (fixed-effect) parameters that characterise each study; however, as individual data are often fitted jointly, estimation of individual-level parameters is influenced by population-level data and assumptions (Cleasby et al., 2015). Revisiting these pillars matters when researching new systems and fields, or types of data, where there is less experience in support of a given working assumption carried over from proved convention in more traditional areas.

A different type of estimation approach, such as populations of models (PoM) (Goaillard et al., 2009; Marder, 2011; Britton et al., 2013; Mirams et al., 2016), has been developed that uses certain criteria to match trait models to data pooling multiple individual observations, and to generate sets of data-compatible model outputs interpreted as predicted interindividual trait variations. Here, a model corresponds to one explanatory parameter point. Further work (Drovandi et al., 2016) translated the PoM approach into a Bayesian framework (Gelman et al., 2013) using approximate Bayesian computation (ABC), a nonparametric (likelihood-free) form of Bayesian inference (Beaumont, 2010). As in the PoM, Drovandi et al. (2016) assumed that within-individual variability was negligible by representing individual traits by point values and fitted a trait model to data pooling individual trajectories, generating population-level distributions of phenotypes. Bayesian inference incorporates parameter uncertainty and correlations, overcoming limitations of point estimation, for example, maximum likelihood estimation (Gutenkunst et al., 2007; Babtie and Stumpf, 2017). This uncertainty will have components from individual phenotypic variation and from data incompleteness and model inaccuracy. However, like most regression frameworks, conventional applied Bayesian inference is parametric (Gianola et al., 2009; Blasco, 2017). Nonparametric approaches, although more difficult to apply, can capture data variation that is not explicitly modelled (Gonzalez-Recio et al., 2008). Bayesian approaches, such as ABC and others (Wood, 2010; Green et al., 2015), were developed for this reason but are usually applied to replace computationally intractable likelihoods in complex high-dimensional problems (Pritchard et al., 1999; Fearnhead and Prangle, 2012; Sunnaker et al., 2013) rather than as an alternative to making distributional assumptions in any problem, simple or complex.

In this paper, we explore a Bayesian framework combining principles from the above that, like Drovandi et al. (2016), offers alternative nonparametric Bayesian inference and, like DHGLM, accounts for variation within and among individuals. As a case study, the approach is applied to ontogenetic growth focusing on low numbers of traits and model parameters. We test the following hypotheses.

- **Hypothesis 1.** ABC can be used as an inferential tool to estimate model trait distributions from individual trait data requiring no explicit residual distribution (i.e., noise) assumptions and no explicit variance-covariance parameters. **Rationale:** Intraindividual and interindividual phenotype variations have often unknown causes and may or may not be well approximated by or involve misspecification of a parametric likelihood. ABC can be used as a generic nonparametric tool (Green et al., 2015) where the noise distribution is tuned by the data. Moreover, parsimony is gained in that no (auxiliary) distributional parameters need to be estimated or guessed. However, in likelihood-based fitting of multiple trait models, a tractable multivariate distribution is required, and an MVN is typically assumed with a dimension-increasing number of variance-covariance parameters (Blasco, 2017).
- **Hypothesis 2.** Individual trait models are best informed when fitted directly to trait observations from a single organism. We propose a framework for scaling-up individual-level parameter distributions to population-level parameter and trait distributions based on the additivity of probability, which is suitably achieved in a Bayesian context. **Rationale:** Pooled trait data from different individuals may not produce patterns (e.g., growth) that could be biologically generated by a single individual (Begall, 1997), limiting the meaning of individual trait model fits. Likewise, fitting multiple individual data sets, allowing individual-specific parametric distributions but doing so jointly, grants mutual influence between individual and population estimates and could yield per-individual trait estimates with limited support from the individual’s specific data, that is, biased by the majority.

In animal breeding, the accuracy of the approach to estimate individual traits without involving multivariate variance-covariance parameters, which will be demonstrated, could lead to improved selection. We will derive the formalism, explain the Bayesian inference methods and measures of model fit, and detail the case studies on empirical and simulated ontogenetic growth. We test the hypotheses by addressing specific questions in several experiments on individuals and populations (**Table 1**).

**Table 1:** Hypotheses underlying the inferential framework and their testing.

	Hypothesis 1 – Individual	Hypothesis 2 – All individuals in a population
Hypothesis	ABC can be used as an inferential tool to estimate model-trait parameters and model-trait distributions from individual trait data, requiring neither strong distributional assumptions nor estimation of (auxiliary) distribution parameters.	Individual-trait models are best informed when fitted directly to trait observations from a single organism. A suitable framework to infer population-level parameter and trait distributions is to scale-up individual-level parameter distributions using additivity of probability.
Questions	Q1.1 Are ABC inferences as accurate as those from likelihood inference? Q1.2 Are they more accurate under incorrect likelihood assumptions?	Q2.1 Does the populations-level distribution provide a suitable representation of the population data? Q2.2 How do the predictions change when fitting the trait model jointly to multiple individual data? The answers to these questions are conditioned by Question 1.
Experiments	Fitting of Gompertz growth model (Equation 21) to one individual’s data set on: 1) One empirical trait, or one simulated trait with normal or skewed deviations (*Comparison of methods to estimate phenotypes – Individual single-trait phenotypes*, **Figures 2****–****6**). 2) Two empirical traits, two simulated traits with MVN deviations (*Individual multiple-trait phenotypes*, **Figures 7****–****8**).	Fitting of Gompertz growth model to every individual in a population (*From individual phenotypes to population phenotypes*, **Figures 9****–****11**): 1) One empirical trait in pigs fit individually; 2) one empirical trait in chickens of two genetic lines; 3) one empirical trait in pigs fitting pooled data.
Testing (results summarised in **Table S1**)	Quality^2^ of individual-level inferences^1^ and agreement between ABC and BL inferences. Sensitivity of ABC and BL inference quality to reduction in data sample size. Sensitivity of BL to likelihood assumptions (Equations 15–17).	Plausibility of population-level inferences and agreement between ABC and BL inferences. Nature of variation in individual traits (parameter mode) and its agreement between ABC and BL approaches. Detection of population heterogeneity (genetic, environmental).
1 Inferences	• Parameter posterior distribution and its mode and uncertainty (range). • Trait predictive posterior distribution and its median and mode curves and uncertainty (range or credible interval)	
2 Quality of the inferences	• Fitting empirical data (Hypotheses 1 and 2): Plausibility of the mode parameters and median and mode trait curves (when a likelihood is used, the mode parameters and mode trait curves are the maximum-likelihood estimates). • Fitting simulated data (Hypothesis 1): Level of match of the expected parameters and trait curve (target). • Fitting empirical or simulated data: Plausible uncertainty and goodness of fit (Equation 20).	

## Methods

### A Probabilistic Framework for Modelling Phenotypes in Individuals and Populations

We consider a given organism and a population of biologically similar individuals. For each individual i in a population of size N (i = 1…N), we consider a phenotype defined by a set of observable traits Ti and a mathematical model Mi of these traits in terms of a given vector of covariates X,

(1) $$M_{\text{i}}=\text{m}(\theta_{i},\theta_{\text{p}},X)$$

where m is a function (representing putative mechanisms shared by every individual), θi is a vector of individual-specific parameters, and θP is a vector of parameters shared by all individuals in the population or in subgroups thereof. The function m may be deterministic or stochastic, but here it is assumed to be deterministic as we do not know enough about the causes of variation within an individual’s trajectory along X to model such variation explicitly. Deviation from the data (for given parameters) is modelled separately (as noise, Equations 15–17) (Hartig et al., 2011). The function m is arbitrary; it may be a linear or nonlinear function of the additive effects of X, as in regression models. Alternatively, m may have any level of complexity through a set of differential or algebraic equations or recurrent procedures defined in an algorithm, that is, m may not be an explicitly tractable mathematical function of X. Parameters θi may be thought of as model-derived phenotypic traits of individual i or as individual-specific conditions, and θP may be associated with population-wide factors, for example, species, genetic line, or diet, or with covariate X, for example, age of a cohort or environmental conditions. This separation of parameters conforms to hierarchical regression models and to mixed-effect models in particular (Hill, 2010; Gelman et al., 2013; Blasco, 2017), where θi and θP play the roles of random-effects and fixed-effects, respectively. We will study examples where Ti comprises live weight and energy intake of an animal at serial time points X, θi represents mature size and time to reach maturity for each of the traits, and θP relates to line, diet, and macroenvironmental conditions [it is implicit that there is nonmeasurable microvariation in environmental conditions among individuals (Hill and Mulder, 2010)].

In a Bayesian modelling framework, where all quantities are described by probability distributions, the traits Ti and the parameters are modelled by probability densities: ƒi(Mi) and πi(飉θi,θP), where Mi, θi, and θP take values over ranges defined by these distributions. Subscript i indicates individual-specific distributions and variables. One useful summary is the expected value of the traits:

(2) $$\text{E}[T_{\text{i}}]=\int M_{\text{i}}f_{\text{i}}(M_{i})dM_{i}=\int \text{m(}\theta_{\text{i}},\theta_{\text{p}},\text{X})\pi_{\text{i}}(\theta_{\text{i}},\theta_{\text{p}})d\theta_{\text{i}}d\theta_{\text{p}}$$

Assuming that the population-wide factors and thus the associated parameter θ_p_ are held constant, it is useful to consider probability densities conditioned on these:

(3) $$\Pi_{\text{i}}(\theta_{\text{i}})=\pi_{\text{i}}(\theta_{\text{i}}|\theta_{\text{p}})=\pi_{\text{i}}(\theta_{\text{i}}|\theta_{\text{p}})/\pi_{p}(\theta_{p})$$

(4) $$F_{i}(M_{i})=f_{\text{i}}(M_{i})/\pi_{p}(\theta_{p})$$

where $\pi_{\text{p}}(\theta_{p})=\int \text{d}\theta_{\text{i}}\pi_{\text{i}}(\theta_{i},\theta_{\text{p}})$ is the marginal probability density of population parameters, and Π and F omit the explicit dependency on the latter. The conditional expectation of the traits becomes

(5) $$\text{E}[T_{\text{i}}\text{|}\theta_{\text{p}}]=\int M_{\text{i}}f_{\text{i}}(M_{i})\text{/}\pi_{p}(\theta_{p})dM_{i}=\int \text{m(}\theta_{\text{i}},\theta_{\text{p}},\text{X})\;\Pi_{\text{i}}(\theta_{\text{i}})d\theta_{\text{i}}$$

**Figure 1:**
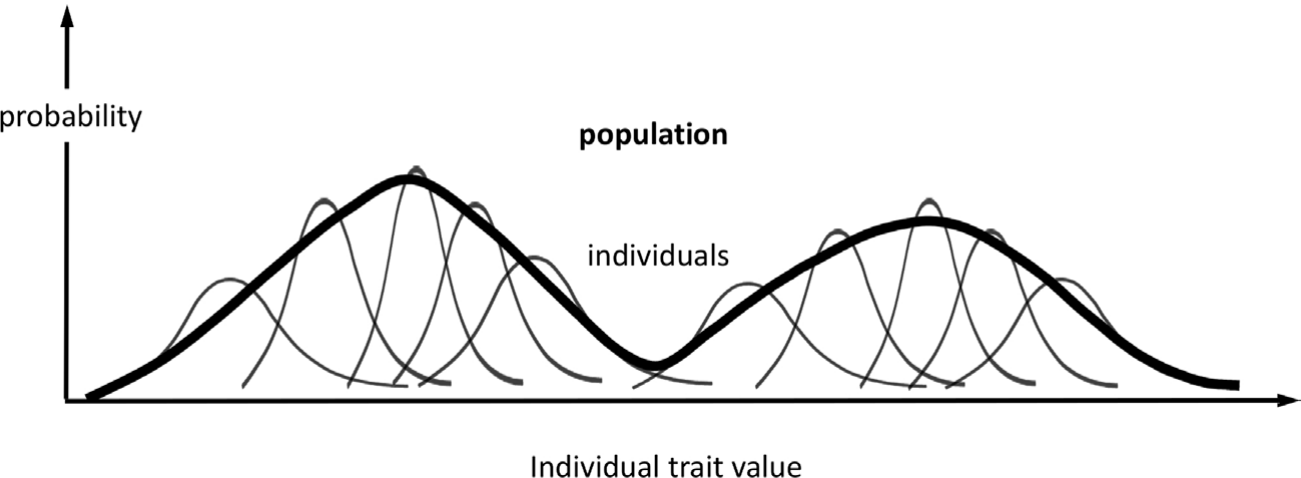
Probabilistic phenotype framework. Schematic representation of the probabilistic framework for modelling phenotype variations in individuals and populations. In this example, the phenotype comprises one trait, and its population distribution is bimodal. The representation of individuals is not exhaustive, and the representation of the population distribution is qualitative.

At the population level, we define distributions associated with the same phenotype (set of traits) as for each individual in the population (**Figure 1**): ƒ({Mi,i = 1…N}) and π({θi,i = 1…N}, θP); and, if we condition on held values of the population parameters, F({Mi,i = 1…N}) = ƒ({Mi,i = 1…N})/πP(θP), and Π({θi,i = 1…N}) = π({θi,i = 1…N},θP)/πP(θP), which, again, omit explicit dependency on θP. We assume that the parameters θi are independent variables among all individuals when conditioned on the same fixed values of the population or group parameters θP. As the parameters θi determine the observed traits Ti, this assumption means that each individual responds independently (according to its genotype, past experience, etc.) to the shared population conditions, such as cohort age or environmental stimuli. While we could treat Π as a product of individual distributions comprising all individual parameters, it is more useful to scale up the traits to population level by combining individual information and missing track of individual sources of phenotypic expression. Hence, we regard every θi as an expression of the same set of traits θ; or, similarly, we regard πi(θi) as an individual’s sample frequency of the population traits θ. Likewise, a population phenotype set of variables (T), qualitatively similar to the individual phenotype (Ti), can be defined and modelled for each given value θ as

(6) $$\text{M}=\text{m}\left(\theta ,\theta_{\text{P}},\text{ X}\right)$$

Hence, the population distribution of model trait parameters is the sum of all individual contributions

(7) $$\Pi \;(\theta )=\text{ A}\underset{\left\{\text{i}=1\ldots \text{N}\right\}}{\sum }w_{\text{i}}\frac{\pi_{\text{i}}\left(\theta ,\;\theta_{\text{P}}\right)}{\pi_{\text{P}}\left(\theta_{\text{P}}\right)}=\text{ A }\underset{\left\{\text{i}=1\ldots \text{N}\right\}}{\sum }w_{\text{i}}\Pi_{\text{i}}\;(\theta ),$$

where the weights wi $\left(\Sigma_{\left\{\text{i}=1\ldots \text{N}\right\}}w_{\text{i}}=1\right)$ quantify relative confidence in the estimated Πi associated with the data volume of each individual (for the data sets we use, wi = 1) and ${\text{A}}^{-1}=\sum_{\left\{\text{i}=1\ldots \text{N}\right\}}w_{\text{I}}\int \text{d}\theta_{\text{i}}\Pi_{\text{i}}(\theta )$. The associated population distribution of modelled phenotypes is:

(8) $$\text{F}\left(\text{M}\right)=f\left(\text{M}\right)\text{/}\pi_{\text{P}}\left(\theta_{\text{P}}\right),$$

and the population expectation of phenotype T is:

(9) $$\text{E}\left[\text{T|}\theta_{\text{P}}\right]=\int \text{M F}\left(\text{M}\right)\text{dM}=\int \text{m}\left(\theta ,\theta_{\text{P}},\text{X}\right)\;\Pi \;(\theta )\text{d}\theta .$$

Other useful summary statistics of this theoretical model-specific phenotype distribution F, such as quantiles and credible intervals, are evaluated similarly.

In this framework (**Figure 1**), we regard πi(톽θi, θP) and ƒi(Mi) as inherent properties of each organism, representing potential intraindividual variability [phenotypical plasticity (Hill and Mulder, 2010)], as well as inferential uncertainty about model parameters and phenotype predictions. On the other hand, we regard π(θ,θP) and F(M) as population-specific properties on the same phenotypes.

### Bayesian Inference

Our next step concerns how to estimate the above theoretical distributions from empirical data on individual traits. Given observation data D ≡ D(θP,X) = {Ti, i = 1…N} under population conditions θP and X, we are interested in estimating the distribution f(M) of modelled phenotypes under a certain phenotype model m. According to Equation 9, we need first to estimate the parameter distribution Π(θ). A posterior distribution (PD) of parameters is obtained from given trait data and a model (relating traits and parameters) through Bayes’ theorem (Gelman et al., 2013):

(10) π(θ|D,m)=L(D|θ,m)π(θ)/C

that combines a prior parameter distribution (assumed by the user) with the likelihood L(D|θ,m) (probability of observing the data given model m and parameters θ). Computation of the constant C=∫L(D|θ,m)π(θ)dθ  requires evaluation across the parameter region where likelihood x Prior > 0, and in nearly all applications can only be carried out through partial sampling of parameter space, for example, *via* Markov-chain Monte Carlo (MCMC) techniques (Gilks et al., 1997).

### Nonparametric (Likelihood-Free) Inference

ABC infers the parameter PD of a model without explicit computation of the likelihood (Beaumont, 2010; Sunnaker et al., 2013). In ABC, multiple model outputs are simulated for sampled parameter points, and these parameters are assessed according to a measure of discrepancy between the data and model outputs. Let Di(θP,X) be the data set for individual i (over a range X ≡ t = {t_1,..,t_ni} of the independent variable, such as age or a varying environmental factor). For each sampled parameter point θ, the model output (or outputs if the model were stochastic) is M(θ); the parameter is accepted if a user-defined distance between model and data is below a certain tolerance ε: d(Di, M(췧θ)) < ε. Often, summary statistics of the data, S(Di), are used instead to reduce dimensionality and computation, with acceptance criterion:

(11) d(S(Di),S(M(θ)))<ε

We chose the following distance function:

(12) $$\text{d}\left(\text{Di},\text{M}\left(\theta \right)\right)=\;\frac{1}{\text{ni}}\sum_{\text{j}=1}^{\text{ni}}\left|\frac{\left[\text{M}\left(\theta ,{\text{t}}_{\text{j}}\right)-\text{Di}\left({\text{t}}_{\text{j}}\right)\right]}{\text{Di}\left({\text{t}}_{\text{j}}\right)}\right|$$

which applies to data Di > 0 and has several benefits: offers automatic relative scaling of model-data discrepancies; being an average relative error, provides immediate interpretation of the tolerance parameter; for example, ε = 0.05 means that parameters θ yielding relative discrepancy up to 5% is accepted. We found that this distance confers faster convergence of MCMC parameter sampling (see below) than a distance with a fixed scaling parameter (in the denominator in Equation 12). The summation over data points (t), a common choice in ABC studies (Toni et al., 2009; Kypraios et al., 2017), implies the constraint acts on the average distance across the data set, allowing outliers to be above tolerance, that is, to have limited influence on fitting, at the cost of better-matching points. To fit data on multiple traits, we applied Equations 11 and 12 to each trait separately and conditioned parameter acceptance to satisfaction of every trait-specific criterion. The above distance relates to but differs from another used by Pritchard et al. (1999). We use a data-based estimate of tolerance for each trait. This estimate is based on the notion that the rate of change in the data partially captures the scale of data random variation and on averaging this rate across points:

(13) $$\varepsilon_{\text{est}}=\frac{1}{\text{ni}}\underset{\text{j}}{\sum }\frac{\tau }{\left({\text{t}}_{\text{j}}-{\text{t}}_{\text{j}-1}\right)}\left|\frac{\left[\text{Di}\left({\text{t}}_{\text{j}}\right)-\text{Di}\left({\text{t}}_{\text{j}-1}\right)\right]}{\text{ Di}\left({\text{t}}_{\text{j}}\right)}\right|$$

where τ = 1 is a dimensional constant. This estimate applies when the amplitude of random variation dominates over trend variation with t, sequential random variation is at most weakly correlated, and when there are frequent observations. We found ε_est_ to be a reasonable guide on the minimum distance to a given data set for the models we fitted; in the case of simulated data, this minimum distance can be calculated exactly, and we found it to be just under ε_est_. Adjustment around this approximation may be necessary to ensure suitable tail off of parameter PDs within the prior parameter range.

We implemented ABC using MCMC sampling (Marjoram et al., 2003). MCMC is more efficient than simple parameter rejection sampling, whereas Sequential Monte-Carlo sampling (Toni et al., 2009) can be more cost-effective in ensuring chain convergence but involves more tuning (Kypraios et al., 2017). For the purpose of this paper, the sampling method is not central and MCMC is efficient enough. Details of the algorithm used are in Text S1. ABC software packages with efficient, widely tested, and user-friendly algorithms are available for general applications (Csillery et al., 2012; Nunes and Prangle, 2015). We used objective uniform parameter priors with ranges guided directly by the data range using the biological interpretation of the model parameters. Parameter PD densities were derived from MCMC samples using a parameter grid. For a parameter θ that relates to the magnitude of trait D, such as mature size (section *Case Study: Ontogenetic Growth at the Individual and Population Levels*), the grid cell size (inverse resolution), δθ, must exceed a minimum so that data-D-driven sample variation about the centre of the cell is predominantly contained within the cell:

(14) $$\delta \theta >2\;\varepsilon \;\theta_{\text{av}}$$

where θ_av_ is an average value (estimated, e.g., from an MCMC sample) and ε is the tolerance used for trait D. Conversely, a maximum tolerance given a grid resolution can be set at ε_max_ = δθ/θ_av_. These relationships give meaning to the tuning of parameters, helping to reduce arbitrariness in their setting.

### Comparison Against Likelihood-Based Inference

To test the performance of ABC, we compared its inferences with those of a Bayesian likelihood (BL) approach. Testing was done on data sets described below, including observation data on ontogenetic growth and simulated data. When fitting observation data, we can assess goodness of fit and apparent plausibility of estimated parameters and traits, but we do not know whether the estimates reflect accurately the underlying processes that originated the data. An inverse testing can be done by fitting simulated randomised data comparable in features and scale to the observation data, but for which we know the generating parameters and model; these generating parameters are, therefore, the target of the inference. A satisfactory inferential approach should estimate parameters close to the target, although random variation and incompleteness in the data prevent exact estimation. Using simulation also allows testing of distributional assumptions; we consider some deviation of the likelihood from the data-generating distribution. In both the ABC and BL approaches, the inferences consist of estimated parameter PDs and trait-predictive PDs. We use the mode of the parameter PDs as point information [known in the likelihood case as maximum likelihood estimate (MLE)] and percentiles of the predictive PDs of the traits [including median and 80% credible interval (CrI)] as distributional information. In the case-study data sets, there is a covariate (time); we evaluated the predictive PDs within and beyond the time range of the data to assess trait forecasting.

In BL inference, the use of uniform parameter priors (the same as those in ABC) implies that the posterior parameter density (π(θ|D,m), Equation 10) coincides with the likelihood up to a normalising constant specific to the prior range. To give a stringent test on ABC by avoiding parameter sample variation in BL estimation, we evaluated the likelihood on a discrete parameter grid, which in low-dimension problems can be accurate and computationally viable. In most applications below, we use normal (or MVN) likelihood functions that assume each data trait has temporally-uncorrelated noise variation with constant variance about a time-dependent model mean:

(15) D(t)−M(t,θ)~N(0,Σ)

where D(t)-M(t,θ) is the vector of deviations between data and model mean for each trait at time t, and Σ is the trait’s noise variance-covariance matrix. In some cases, we assess the impact of likelihood assumptions on inference by considering a commonly found form of heterocedasticity, known as multiplicative noise, where the variance of a trait increases with the magnitude of the trait variable:

(16) $$\left[\text{D}\left(\text{t}\right)-\text{M}\left(\text{t},\theta \right)\right]{\text{ M}}^{-1}\;\sim \text{N}\left(0,\Sigma \right)$$

where M^−1^(t,θ) is a vector whose elements are the inverse means for each trait at given t, θ. The point of exploring the sensitivity of BL inference to distributional assumptions is that ABC captures the data noise distribution automatically (up to a point). In addition, to test the ability of ABC and likelihood to deal with skewed data, we consider an extreme case where data are totally negatively skewed

(17) $$\left[\text{D}\left(\text{t}\right)-\text{M}\left(\text{t},\theta \right)\right]{\text{ S}}^{-1}\sim -\;\sigma \;\chi_{1}$$

that assumes zero trait correlations and has variance 2σ^2^; we use σ = 0.05, which yields large multiplicative variations (14% multiplicative reduction at two standard deviations in Equation 17). Here, S = M(t,θ) or 1. This example is motivated by ontogenetic growth, where some temporal variation may result from short-term limitation in availability of resource and thus in downward deviation. Observation data (described below) were fitted using likelihoods associated with the different variability assumptions (Equations 15, 16) as well as using ABC. Simulated data were generated by superimposing on the trait model M(t,θ) temporally uncorrelated noise sampled *via* Equation 15 or 17. These data were fitted using ABC or BL with the likelihood assuming the same noise model that generated the data (Equation 15 or 17) or a slightly erroneous noise distribution (Equation 16). The likelihoods associated with Equations 15 and 16 involve additional unknown variance and covariance parameters. For a phenotype specified by a single trait, the additional parameter is a variance; in the case of two traits, there are two variance parameters and one correlation parameter. We used initial estimates of variance based on a scaling relationship (see below) and estimates of correlation based on the data; uniform prior distributions about these estimates were used for these auxiliary parameters in Equations 15 and 16, assuming a temporally constant variance ratio in the case of multiple traits. The BL parameter PD used for comparison with ABC is marginal distribution obtained *via* integration on the auxiliary parameters in the likelihood.

#### Relationship Between Scales of Trait Variation in ABC and Likelihood Inference

In ABC inference, there are tuning parameters that partially substitute the role to a variance-covariance matrix in either BL or maximum likelihood inference. The most influential, apart from the distance function, are probably the tolerance parameters (Kypraios et al., 2017). The number of such parameters is similar in the ABC and BL approaches for low-dimensional problems, but, as dimensionality increases, the number of likelihood auxiliary parameters increases faster, as n(n + 1)/2 for n variables. The likelihood variance parameter (element σ of Σ) and ABC tolerance parameter (ε) associated with a trait can be related as they represent, respectively, the average deviation and the average relative deviation (Equation 12) between model and data. Hence, in the spirit of Equation 14, we expect that:

(18) $$\sigma =\text{B }\theta_{\text{av}}\;\varepsilon$$

where θ_av_ (defined in Equation 14) is the average magnitude of the trait; ε = ε_est_ (Equation 13), and B is a proportionality factor. We used Equation 18 to set a guide value for the unknown variances in Σ, allowing a fair comparison between ABC and BL PDs with least reliance on arbitrarily independently-set scales of trait variation. We used a uniform prior on B with range [0.5,2].

### Evaluation

To assess overall model fit (how well the model replicates the data), we use the familiar coefficient of determination R^2^ (proportion of variation in the data explained by the model) as an absolute measure of fit [as in other ABC studies, e.g., van der Vaart et al. (2015)]. R^2^ is adjusted for the number of parameters by scaling the data and model variances by the respective number of degrees of freedom, dfD and dfM,

(19) $${\text{R}}_{\text{adj}}\;=1-(1-{\text{R}}^{2})\frac{{\text{df}}_{\text{D}}}{{\text{df}}_{\text{M}}}$$

Following standard Bayesian definition of test statistics (Gelman et al., 2013), we use a Bayesian extension of R_adj_^2^ that sums squared residuals, between model point prediction and data, weighed by the estimated parameter PD:

(20) RBayes,i2=1−ϕ∫​dθ∫​dM∑t=1ni(Di(t)−M(t))2f(M|θ,D)πi(θ|D)∑t=1ni(Di(t)−mean(Di))2RBayes,i2= 1−ϕ∫​dθ∑t=1ni(Di(t)−M(t,θ))2πi(θ|D)∑t=1ni(Di(t)−mean(Di))2

Here, ϕ = dfD/dfM. The right-hand side applies to deterministic models, where there is a single model prediction for given parameters (f(M|θ,D) = δ(M-M(θ)). Index i indicates that variability is intraindividual i, and index Bayes distinguishes this R^2^ from usual estimates based on parameter point estimates. We will drop index notation i when a single individual is considered. Note that, strictly, this statistic is not comparable among cases where different data are used. R_Bayes_^2^ is a summary of model residuals that does not account for potential trend; to assess qualitative fit and (to some extent) the predictive potential of the model, we use visual diagnostics of predictive PDs (Gelman et al., 2013) and, in **Figure 3**, examine their associated distribution of residuals across the fitted data. In addition, we extend prediction beyond the range of the data’s covariate (time) to show the extent of increase in uncertainty. Although the fit of data by predictive PDs can be assessed quantitatively *via* p-values on suitable test statistics, their interpretation is less clear than that of classical p-values (Gelman et al., 2013) and is even less established within ABC methods (Beaumont, 2010; Lintusaari et al., 2017). When using simulated data, a p-value could be defined on the distance between model and target, a statistic not used in model fitting; this was unnecessary for the study cases below because the inferences obtained were clearly close to the target.

### Case Study: Ontogenetic Growth at the Individual and Population Levels

We use ontogenetic growth as an example application; we focus on individual live weight and energy intake and specify mathematical models and empirical data for these traits.

#### Body Weight Model

It is common, for example, in animal genetics (Emmans and Kyriazakis, 1997; Coyne et al., 2017), to use a parametric curve to describe observed growth trajectories of individual organisms. The interpretation of the curve is that the information contained in its parameters offers a low-dimension summary of serial and noisy data difficult to characterise per se. By assuming that a single growth curve applies across individuals and populations, the variation in model parameters offers a way of capturing interindividual and intraindividual variation in growth. The curve parameters may, therefore, be regarded as non-directly-observed traits that can be compared across individuals, populations, and species. We use a Gompertz model (Winsor, 1932) to describe individual growth trajectories in terms of size, that is, mass (M) measured through body weight, over time (t):

(21) $$\text{M}\left(\text{t}\right)=\text{ K }\exp\left(-\text{a}\exp\left(-\frac{\text{t}}{\text{b}}\right)\right)$$

where K and b are the mature size and a maturation timescale; a = ln(K/M_0_), and M_0_ = M(0) is size at a reference age when t = 0. It is usual to think of u(t) = M(t)/K as a degree of maturity, which leads to:

(26) $$\frac{\text{M}\left(\text{t}\right)}{\text{K}}=\;{\left(\frac{{\text{M}}_{0}}{\text{K}}\right)}^{\exp\left(-\frac{\text{t}}{\text{b}}\right)},\text{ or},\text{ u}\left(\text{t}\right)=\text{ u}{\left(0\right)}^{\exp\left(-\frac{\text{t}}{\text{b}}\right)}$$

where b is the age when log maturity has changed by 1/e, with e being the base of natural logarithms. Among the multiple sigmoidal growth models in the literature, the Gompertz model offers a good balance between simplicity and applicability (Wellock et al., 2004), and its parameters are biologically interpretable.

#### Energy Intake Model

To extend the modelling and estimation to individual phenotypes defined by two traits, we consider energy intake and body weight as an example of correlated traits. Here, for simplicity, we consider energy intake to be also a Gompertz function of age, which captures increase and ultimate levelling-off of intake, although other suitable sigmoidal functions, often of body weight, have been used (Parks, 1982; Black, 2009). Our choice aims at using data to inform trait models without necessarily using previous knowledge, and the fact that age has smaller measurement errors than weight. Feed intake was converted to energy intake by multiplying by diet energy content; net energy is used below. As for body weight, it is assumed that the same energy intake model applies across individuals.

### Data

To demonstrate the approaches to modelling phenotype variability, we use data on one or two traits. Data availability: All data analysed in this study are available online through an institutional data repository (Filipe et al., 2018).

#### Empirical Data

We use serial observations of live body weight (BW) and net-energy intake (NEI) of individual growing pigs and broiler chickens; their specific genetic lines are not relevant for the purposes of the paper. These data cover the typical period of postweaning or post-hatch growth of livestock and may or may not include the stage of change from accelerating to decelerating growth. To demonstrate how the approach scales up from individual distributed phenotypes to population-wide phenotypes, we consider empirical data from multiple individual single-line pigs and from broilers of two distinct lines.

#### Simulated Data

The generation of artificial data was designed to produce data with ranges and trends comparable (but not identical) to those of the observed data and associated parameters of fitted growth curves. As explained above, the main purpose of fitting such data is to indirectly assess the accuracy of the model parameters inferred when fitting observation data, provided the model is a meaningful representation of the processes that generated the observations and thus that such accuracy is possible. Simulated data were generated in two steps: 1) Choosing a function and parameters to represent the temporal trend in each trait; that is, target parameters of the Gompertz curves for BW, (K,b) = (100,50), and NEI, (K2,b2) = (25,30). 2) Overlaying on the temporal trend random noise sampled from a normal distribution, for a phenotype characterised by a single trait, or from an MVN distribution for a phenotype characterised by multiple correlated traits (here, two). The noise model in Equation 15 corresponds to zero-mean constant-variance noise and is a standard choice in literature; Equation 16 corresponds to mean-one multiplicative noise and is based on the observation that variation in ontogenetic growth tends to increase with size (Coyne et al., 2017). Equation 17 corresponds to skewed multiplicative noise and is used to assess the ability to cope with strong deviations from normality; for example, traits associated with heath status, including feed intake, can exhibit skewed variation. The ability of ABC inference to capture standard and nonstandard data distributions can be tested by fitting data generated from given distributional assumptions (Equations 15–17). Likewise, the likelihood inference can be tested for robustness to change in distributional assumptions.

## Results

We test Hypotheses 1 and 2 by addressing Questions 1 and 2 on individuals and populations, respectively, and by using the experiments and testing specified in **Table 1**.

### Comparison of Methods to Estimate Phenotypes: Individual Single-Trait Phenotypes

#### Body Weight of Individual Pig

**Figure 2:**
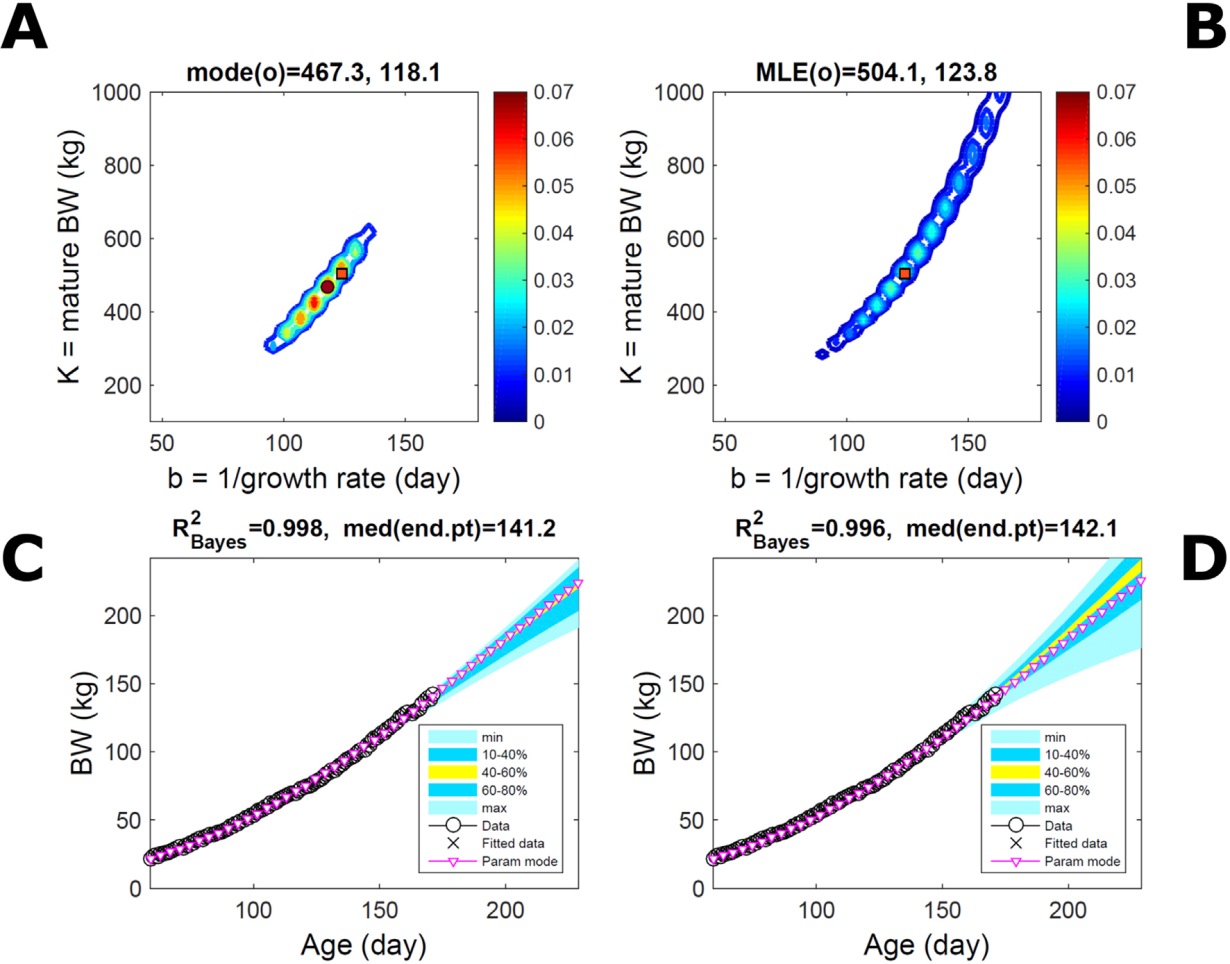
Body weight of individual pig. Trait parameters and temporal distribution estimated *via* ABC (left) and additive-normal likelihood (right) (Equation 15). **(A, B)** Posterior distribution (PD) of the parameters of the Gompertz model (Equation 21); K, mature body weight, and b, time to maturity); colour scale represents probability density. **(C, D)** Predictive PD of body weight; colour represents cumulative probability within the quantile ranges in legend (median curve is the centre of the yellow range; 80% credible interval is the range 10–90%). Trait uncertainty within the data range is caused by temporal variation (e.g., measurement error or animal status) or insufficient data (i.e., multiple curves explaining the data); beyond the data range (>180d), uncertainty about the predicted trait increases considerably. The mode curve (∇) refers to the Gompertz model with parameters given by the mode of the parameter posterior [ABC: white circle (ο) in **A**; likelihood: white square (◻) in **B** and **A**], which differs from the predictive posterior median unless distributions are symmetrical. The predictions based on standard maximum likelihood estimation are the parameter mode (in **B**) and mode curve (in **D**). R^2^ is a Bayesian version of the goodness-of-fit statistic (see Methods).

**Figure 3:**
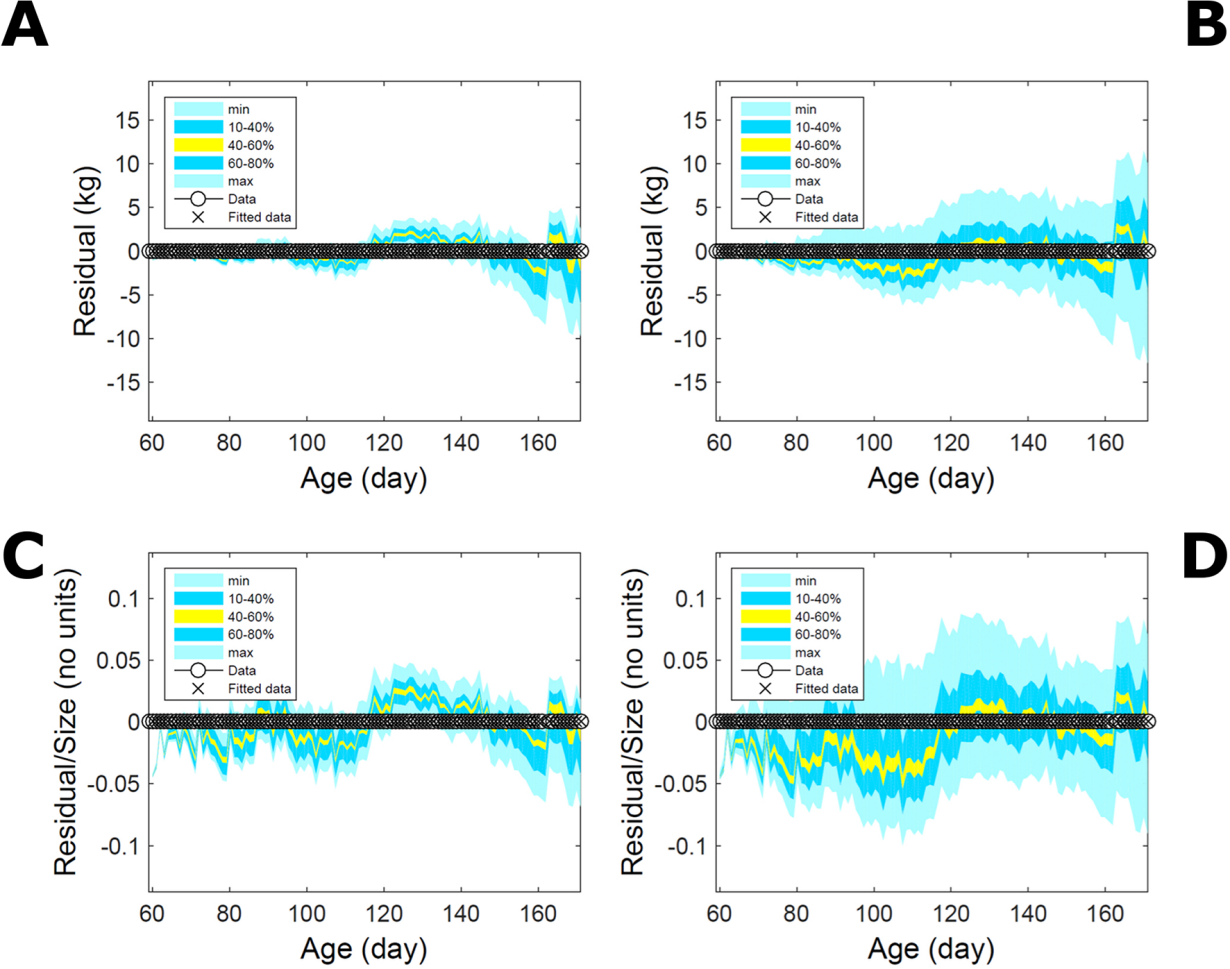
Body weight of individual pig: residuals. Residual difference between the fitted data (110 points) and growth model (Equation 21) fitted in **Figure 2***via* ABC (left) and *via* additive-normal likelihood (right). **(A, B)** Absolute residuals. **(C, D)** Residuals scaled by observation. Colour shows cumulative probability within the quantile ranges in the legend (median is the centre of the yellow range).

**Figure 4:**
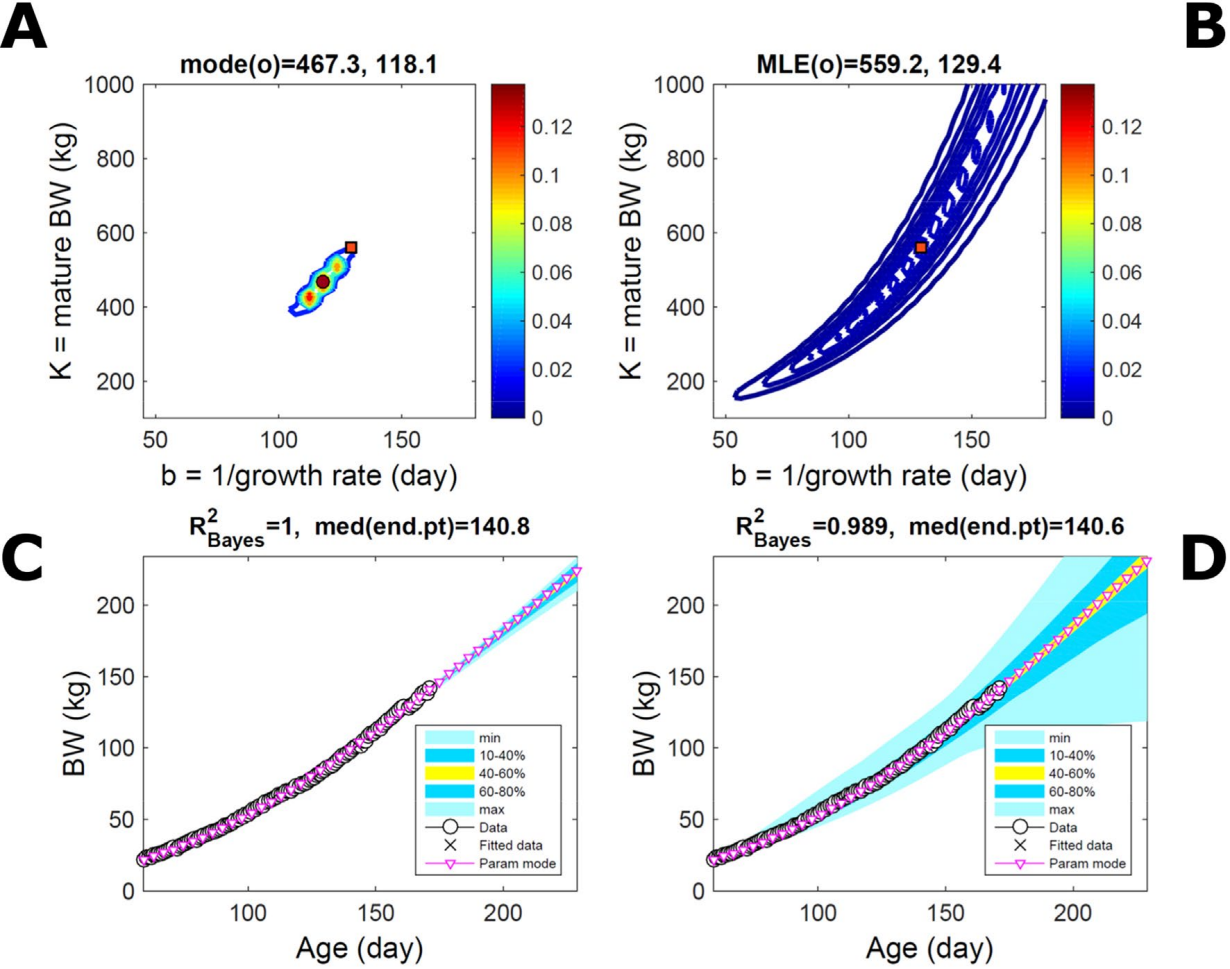
Body weight of individual pig: effect of reduced data volume. Trait parameters and temporal distribution estimated *via* ABC (left) and normal likelihood (right) as in **Figure 2**, but fitting 7/110 data points (6%). **(A, B)** Parameter posterior distribution (PD) of the Gompertz model. **(C, D)** Predictive PD of body weight (BW). Other specifications as in **Figure 2**.

Fitting the model (Equation 21) to empirical body weight (BW) from one individual pig, we find the parameter posterior distributions (PD) estimated *via* ABC and normal BL (Equation 15) are similar and have similar modes, but there are also differences (**Figures 2A, B**). The ABC and BL parameter modes (**Table S1**) and the ABC parameter uncertainty have plausible values [(Strathe et al., 2010), albeit comparison involves different animal breeds and estimation methods]. The BL PD is wider because of uncertainty about the dispersion parameter [whose prior distribution (Equation 18) is integrated over]. The BW predictive PDs agree closely with the data in both approaches (**Figures 2C, D**, **Table S1**). Beyond the data age range (170d), the two approaches, but BL in particular, predict considerable increases in uncertainty (80% CrI) but yield distinct median curves (centre of yellow area); the BL median curve is above the mode curve (with parameters given by the parameter posterior mode) because of parameter PD skew (**Figure 2B**). The distributions of BW residuals in the ABC and BL approaches do not indicate bias, but their variance increases with size as scaling to size gives a temporally more stable residual amplitude (**Figure 3**). Following this hint on data heterocedasticity and assuming a likelihood for multiplicative noise (Equation 16) shifts the MLE from above to below the ABC mode (**Figure S1**, **Table S1**) and alters the parameter and predictive distributions, but without marked change in the median BW curve and in R^2^. Another relevant factor is data volume. The number of data points fitted, 110, is large as the data comprise daily BW records. Fitting only 7/110 = 6% of the data set (**Figure 4**) does not change goodness of fit (i.e., within-data predictive distribution and R^2^) but alters the PDs: With ABC, the estimated mode is unchanged; with BL, the mode shifts above the ABC mode (**Table S1**) and parameter uncertainty increases overall. The BL uncertainty in BW increases markedly particularly in the out-of-sample prediction. The ABC PDs, however, show reduced uncertainty as estimation is informed by the observed variation (Green et al., 2015), whereas in BL, the uncertainty is assessed in relation to an assumed form of data variation. These examples show sensitivity of likelihood-based inference to data distributional assumptions and data volume, whereas the ABC approach does not require the assumptions and was more robust to change in data volume.

#### Simulated Normally-Distributed Trait

**Figure 5:**
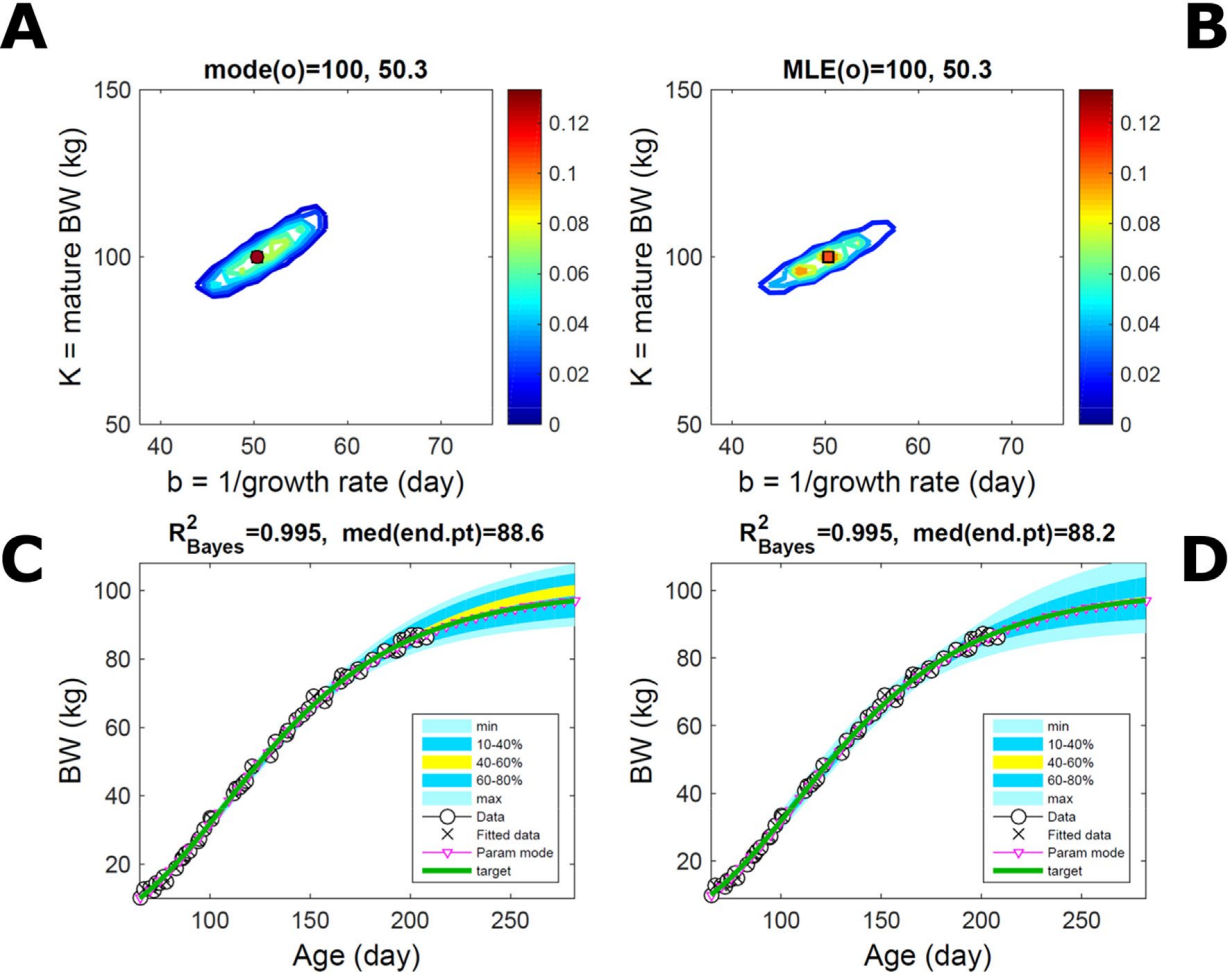
Simulated normally-distributed individual trait. Trait parameters and temporal distribution estimated from simulated individual data, with Gompertz trend (Equation 21) and additive-normal noise (Equation 15), using ABC (left) and additive-normal likelihood (right). Target parameters: (K,b = 100,50). **(A, B)** Parameter posterior distribution (PD) of the Gompertz model. **(C, D)** Predictive PD of body weight. Data comprise 50 points at randomly distributed ages within the given range; noise has standard deviation 1 (four times that of the pig data). Other specifications as in **Figure 2**.

Fitting Equation (21) to simulated growth with additive-normal noise (Equation 15) leads to similar parameter PDs *via* the ABC and additive-normal-BL approaches (**Figures 5A, B**) that predict the parameter target accurately (**Table S1**). This case is a test to ABC but not to the BL approach, which incorporates the exact data distribution and should perform optimally, whereas ABC utilises the data to estimate this distribution as well as (like BL) the model parameter distribution. Compared to **Figure 2**, the ABC parameter distribution has mildly different skew, whereas the BL’s is narrower because it assumes the exact data distribution. The trait’s predictive PDs from both approaches agree closely with the data (**Figures 5C, D**; **Figures S2**, **Table S1**); their spread is similar but the ABC’s uncertainty is slightly wider. Beyond the data range, the BL’s predicted median curve matches the target, whereas the ABC’s slightly overestimates the target (because of the parameter skew), but this is well within the predicted CrI. Accurate prediction of target parameters (and corresponding trait curve) *via* the mode of the PD depended on accord of the parameter grid resolution with Equation (14); too much resolution led to unsuitable sampling and inaccuracy. Giving BL less advantage by assuming a data distribution (multiplicative noise, Equation 16) slightly different from the data-generating distribution led to parameter and predictive PDs identical to those of ABC (figure not shown).

#### Simulated Skew-Distributed Trait

**Figure 6:**
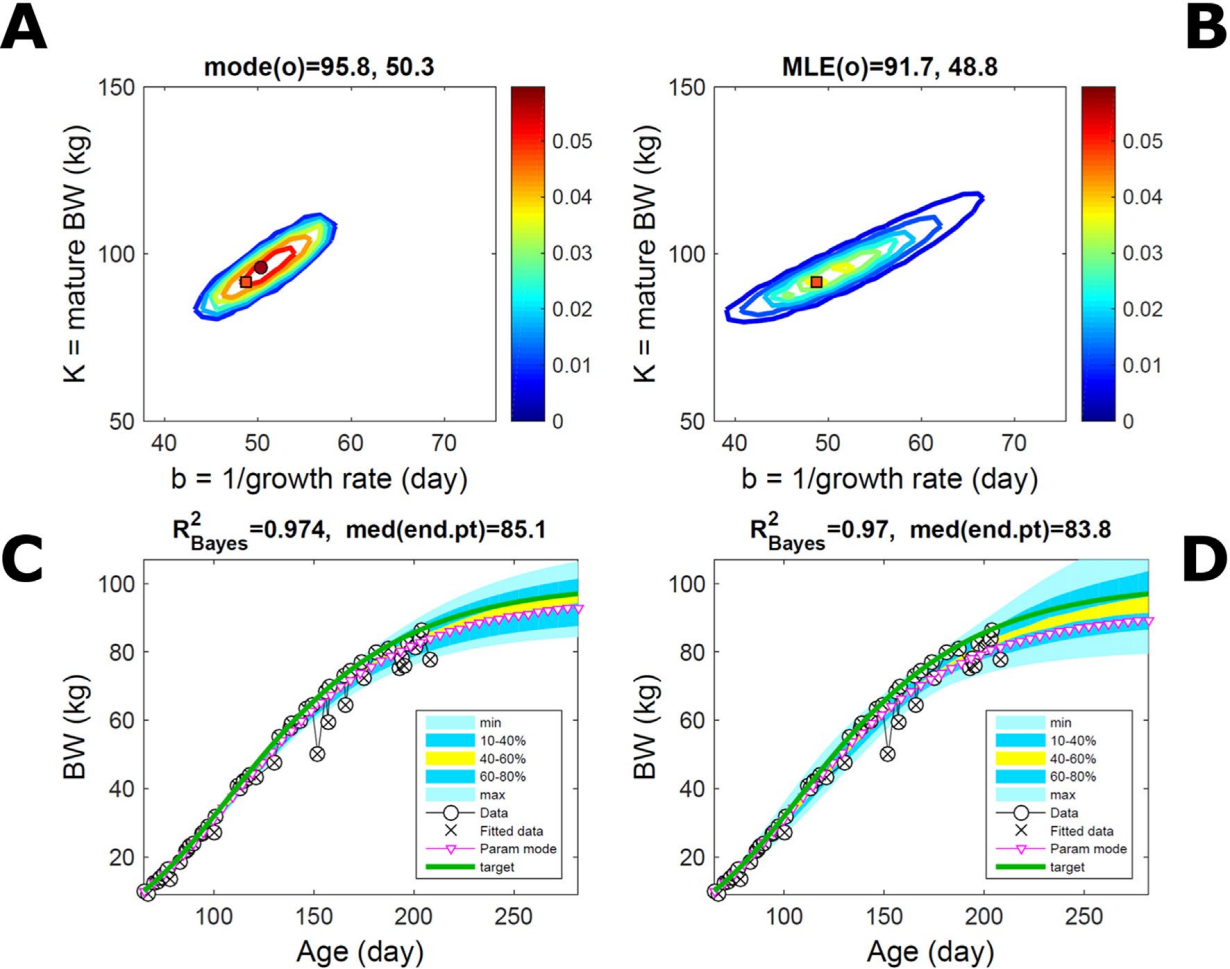
Simulated skew-distributed individual trait. Trait parameters and temporal distribution estimated from simulated individual data with Gompertz trend (Equation 21) and chi-square multiplicative noise (Equation 17), using ABC (left) and normal likelihood (right). Target parameters: (K,b = *100,50*). **(A, B)** Parameter posterior distribution (PD) of the Gompertz model. **(C, D)** Predictive PD of body weight. Data comprise 50 points at randomly distributed ages within the given range; noise has standard deviation 7% (deviations can be two to three times larger). Other details as in **Figure 2**.

Fitting Equation (21) to simulated trait growth with skewed multiplicative noise leads to somewhat different inferences *via* ABC and *via* an (incorrect) additive-normal-BL approach (**Figures 6A, B**). The BL’s prediction of the parameter target is considerably less accurate (**Table S1**), and its parameter uncertainty is wider than the ABC’s. This case is a test to both approaches as none has suitable *a priori* information about the simulated data, which has strong skewed noise, so a close match of the target by either approach is unlikely. The trait predictive PDs fit the simulated data well in both approaches (**Figures 6C, D**; **Figure S3**, **Table S1**). Beyond the data range, the ABC’s predicted median curve and uncertainty (similar to **Figure 5C**) had, respectively, greater accuracy and narrower width than those of the BL approach. Assuming a likelihood closer to the data-generating distribution, that is, multiplicative noise (Equation 16), led to predictions (figure not shown) closer to the target and of similar accuracy to ABC’s, although overall with larger estimated uncertainty; however, ABC did not require this knowledge.

### Individual Multiple-Trait Phenotypes

#### Body Weight and Energy Intake of Individual Pig

**Figure 7:**
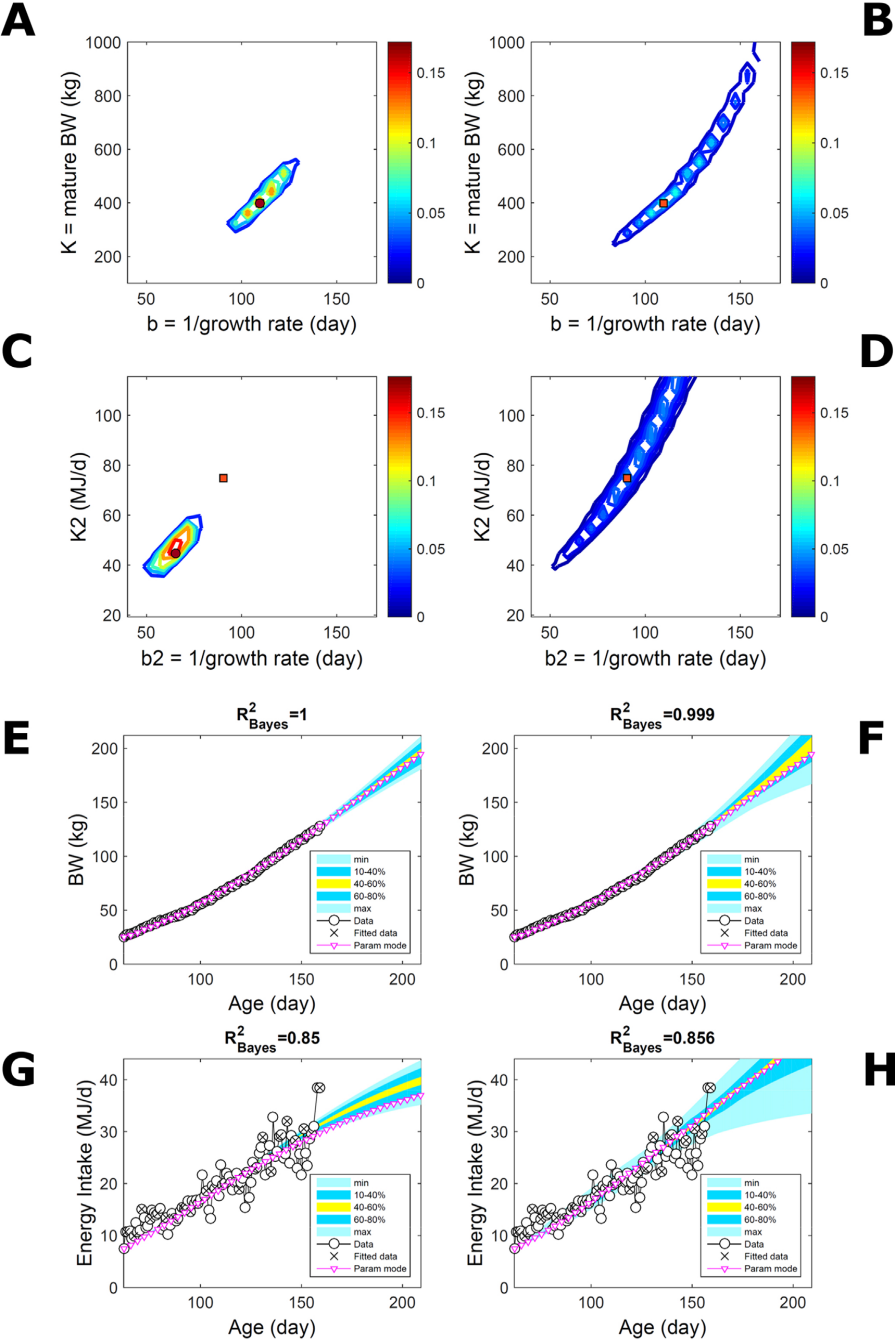
Body weight and energy intake of individual pig. Trait parameters and temporal distribution estimated from two observed correlated traits of the same individual, using ABC (left) and additive-MVN likelihood (right) (Equation 15). Only 25% of the data set is fitted (**Figure S4** shows a fit to the full data). **(A, B)** Marginal parameter posterior distribution (PD) for the body weight (BW) Gompertz model (Equation 21); K, mature size; and b, time to maturity; **(C, D)** likewise for the NEI Gompertz model (K2, mature size; and b2, time to maturity); colour scale as in **Figures 2A, B**; **(E, F)** predictive PD of BW. **(G, H)** likewise for predicted NEI; colour key as in **Figures 2C, D**. The mode curve (∇) refers to the Gompertz model with parameters given by the mode of the parameter posterior [ABC: white circle (ο); likelihood: white square (◻)]. R^2^ is a Bayesian version of the goodness-of-fit statistic (see Methods). Other details as in **Figure 2**.

Fitting Gompertz curves, with parameters K,b and K_2_,b_2_, jointly to empirical BW (as in **Figure 2**) and energy intake (NEI) of one individual pig poses greater inferential challenge because of the larger relative variation in the second trait and the larger number of parameters estimated. Agreement between the ABC and additive-normal-BL inferences depends on the volume of data fitted. Fitting 25% (24/96) of the data set using either approach leads to marginal PDs of BW parameters (**Figures 7A, B**) similar to those in **Figures 2A, B** (except for a sampling-driven reduction in grid resolution). The BW parameter mode is the same using ABC or BL inference (**Table S1**) but differs from **Figure 2**, which refers to a different animal. However, the NEI marginal parameter PDs and their modes (**Figures 7C, D**; **Table S1**) were statistically distinct between approaches because they had very limited overlap. Fitting the whole data set yielded NEI parameter PDs and modes similar between approaches (**Figures S4C, D**) and to those obtained *via* the ABC subset-data fitting (**Figure 7C**). The BW predictive distribution was unaffected by data change in either approach (**Figures 7E, F**; **Figures S4E, F**), apart from a minor shift in the median due to a shift in the parameter distribution (**Figures S4A, B**). The NEI predictive distribution was also largely unaffected by data change when using ABC but was clearly affected when using normal BL (**Figures 7G, H**; **Figures S4G, H**). Here, the change in the NEI mode curve is less meaningful than the change in predicted quantiles, for example, median, as parameter mode estimates are more sensitive to large random variations such as that in the NEI data (as are the estimates of R^2^ and uncertainty). **Figure 7** shows BW and NEI are predicted to reach maturity on *distinct* timescales. These results suggest that the ABC prediction has some accuracy because it is also supported by BL when all data are used.

#### Simulated Correlated Traits With Multivariate-Normal Noise

**Figure 8:**
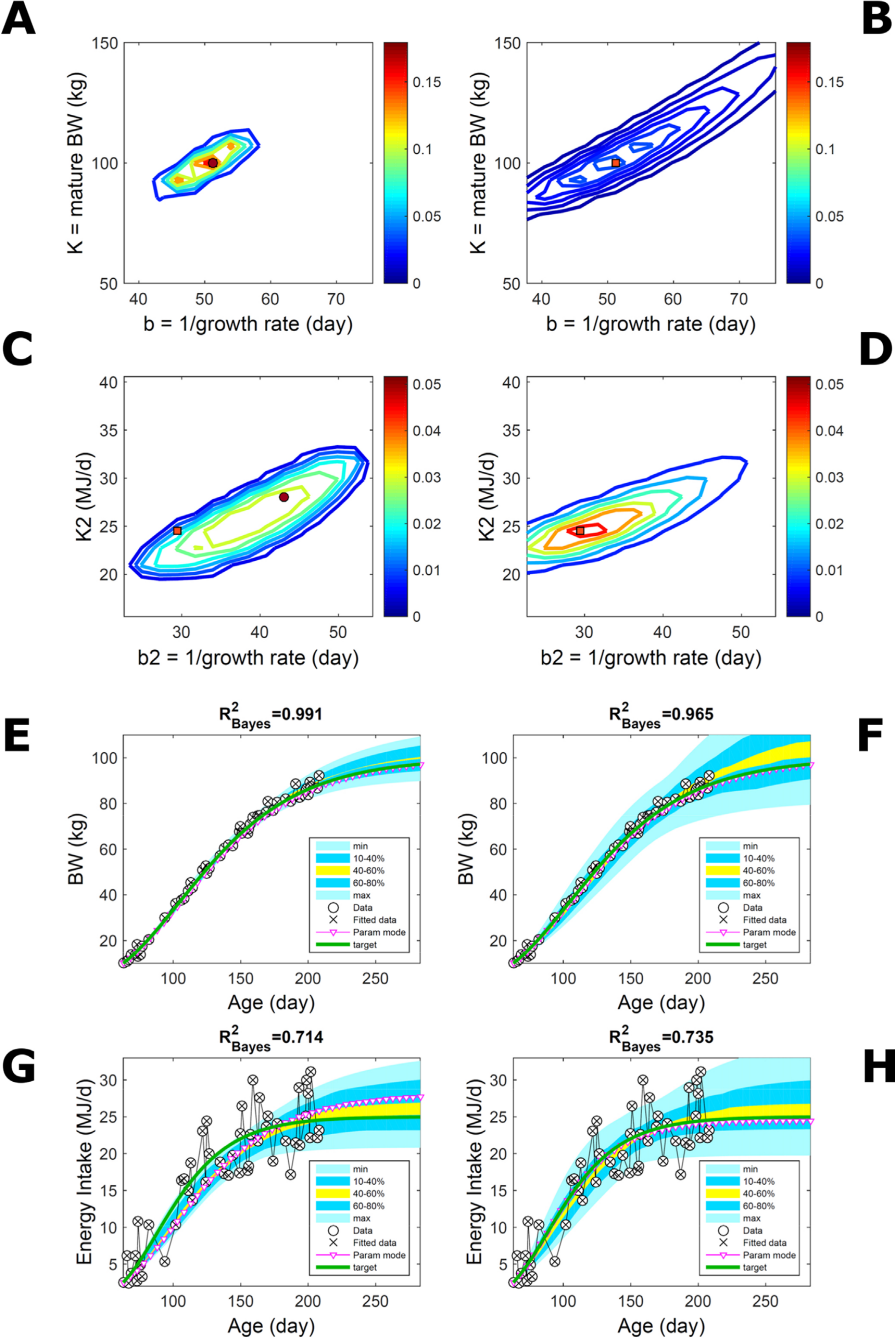
Simulated correlated traits with multivariate normal noise. Trait parameters and temporal distribution estimated from simulated data on two correlated traits of the same individual (Gompertz trend, Equation 21, superimposed with MVN noise, Equation 15), using ABC (left) and additive-MVN likelihood (right) (Equation 15). Target parameters: (K,b) = (100,50) for trait 1, (K2,b2) = (25,30) for trait 2. **(A, B)** Marginal parameter posterior distribution (PD) for Gompertz model of trait 1 (K, maturity limit; and b, time to maturity); **(C, D)** likewise for Gompertz model of trait 2 (K2, maturity limit; and b2, time to maturity); colour scale as in **Figures 2A, B**; **(E, F)** predictive PD of trait 1.**(G, H)** likewise for predicted trait 2; colour key as in **Figures 2C, D**. Data comprise 50 points at randomly distributed ages within the given range; the MVN noise has variance-covariance (var corr; corr var2) = (2^2^ 0.1; 0.1 4^2^).

Two traits with a Gompertz trend are correlated *via* trend if their parameter ratios (K/b) allow the traits to increase similarly within the data range despite random variation. This was the case of the empirical data (**Figure 7**). To extend the simulated data of **Figure 5** to two traits, T1 and T2, we overlaid additive-normal noise (Equation 15) with given variance-covariance onto Gompertz curves with parameters K,b and K_2_,b_2_ with given target values (**Table S1**). This case, like **Figure 5**, is a test on ABC but not on the BL approach, which inputs the exact data distribution (and suitable covariance estimates) and should perform optimally for the given noise. Fitting Gompertz curves to the simulated traits yields largely similar parameter PDs *via* the ABC and additive-MVN-BL approaches (**Figures 8A–D**, for pairwise-marginal distributions, **Table S1**). For T1, the target is predicted accurately by either approach, as in **Figure 5**, although the BL’s parameter distribution is wider (**Figure 8B**). For T2, the target is predicted less accurately by ABC, whose parameter distribution is wider due to the large relative noise in the data but includes the target and its centre is closer to the target than its mode (**Figure 8C**). The T1 predictive-PD of either approach agrees with the data (**Figures 8E, F**), and beyond the data range agrees with the target curve, although the BL’s distribution has larger uncertainty and a median above target because of parameter overspread. The T2 predictive-PD of either approach also agrees with the data in relation to its variation (**Figures 8G, H**); beyond the data range, the medians agree with the target (with the ABC mode curve above target because of mode eccentricity, **Figure 8C**). The ABC T2 distribution does not agree with the target at lower ages, where additive noise disturbs the data disproportionally, causing overestimation of the timescale b_2_, but it agrees with the data. These results show that, despite the extreme random effects in the data and the absence of prior information on these effects, the ABC inferences could capture the overall variability in the data.

### From Individual Phenotypes to Population Phenotypes

Here, we scale up estimation to populations to examine phenotype variation among individuals and characterise phenotypes at the population level. The population-level distribution incorporates variation among and within individuals and uncertainty.

#### Body Weight in a Pig Population

**Figure 9:**
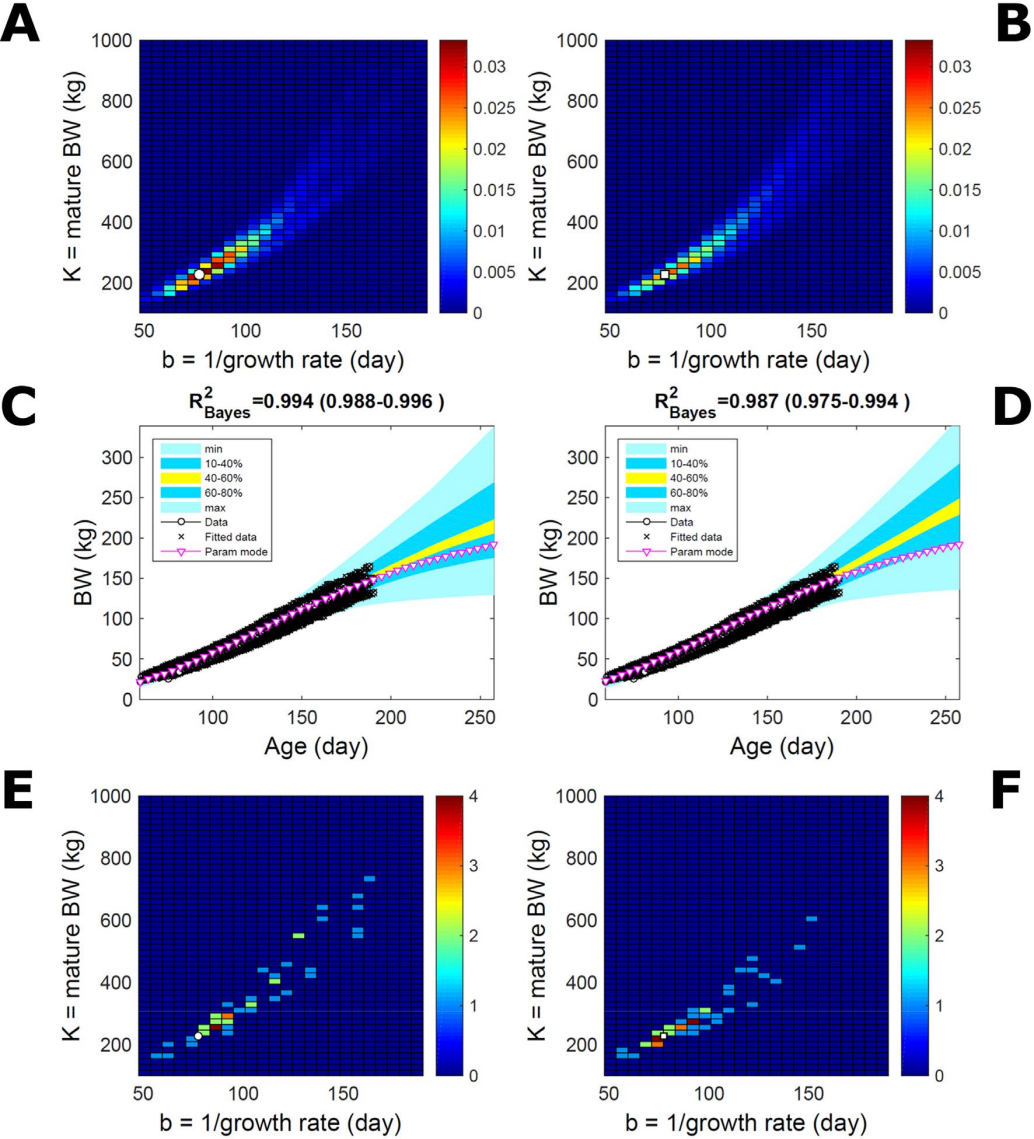
Body weight in a pig population. Estimation *via* ABC (left) and multiplicative-normal likelihood (right). **(A, B)** Population parameter posterior distribution (PD) of the body weight (BW) Gompertz model. **(C, D)** Population predictive PD of BW. **(E, F)** scatterplot of individual mode parameters (K,b); colour scale indicates number of individuals with parameter mode within each cell (the total number of modes equals the number of individuals). Data set with 48 individuals, including those in **Figures 2** and **5**. Other details as in **Figure 2**.

Estimating the PD of Gompertz parameters for BW (Equation 21), as in **Figure 2**, for every individual within a pig population and using the probabilistic framework (see Methods) generates a population PD of parameters. This hyperdistribution refers to individual parameters as it pools individual-level probabilities (Equation 9), and not to parameters of an average individual as would result from fitting pooled data (c.f. **Figure 11**). As the parameters of the BW model are often interpreted as traits, a point (K,b) in parameter space represents a phenotype, and the parameter probability distribution represents the phenotype landscape of the population. We fitted BW data on a group of pigs drawn randomly from a population with unspecified genetic and phenotypic heterogeneity; our purpose is to assess how the ABC and BL approaches characterise heterogeneity. The parameter distributions estimated *via* ABC and multiplicative-normal BL (Equation 16) are similar and have identical modes (**Figures 9A, B**; **Table S1**), quantitatively different from but including the previous individuals (**Figures 2** and **7**). This mode is the most likely phenotype in this population, but is not much more likely than neighbour phenotypes in the landscape; nor is it average as the landscape is skewed. The population BW predictive distributions of the ABC and BL approaches are similar and fit the data well (**Figures 9C, D**; **Table S1**). Beyond the data range, BL predicts a higher median BW and larger uncertainty because of a heavier parameter distribution tail. Although the approaches’ inferences are largely similar at the population level, they differ at the individual level. One way of tracking the phenotype prediction of individuals is to scatterplot their parameter modes (**Figures 9E, F**). This plot shows outlier individuals that are either phenotypically distinct, or misfit by the model due to noisy or perturbed growth trajectories; the key difference between approaches (**Figures 9E, F**) is that ABC predicts more outliers [e.g., mature BW > 550 kg, estimated to be large in Strathe et al. (2010) (p. 644)]. Using additive-normal BL changes the population distributions slightly in relation to the multiplicative-normal BL. The parameter distribution shifts (**Figures S5A, B**; **Table S1**) and so does the BW mode curve, but not the BW predictive-PD (**Figures S5C, D**). The key change is in the pattern of mode scatter (**Figures S5E, F**), with greater mode clustering, around 300 kg, distancing further from the pattern obtained *via* ABC. The consistency between outcomes of the ABC and multiplicative-normal-BL approaches suggests that these outcomes may be the more accurate. Only some individuals had mode predictions affected by change in likelihood, which suggests that individuals may have specific trait distributions.

#### Body Weight in a Mixed-Line Chicken Population

**Figure 10:**
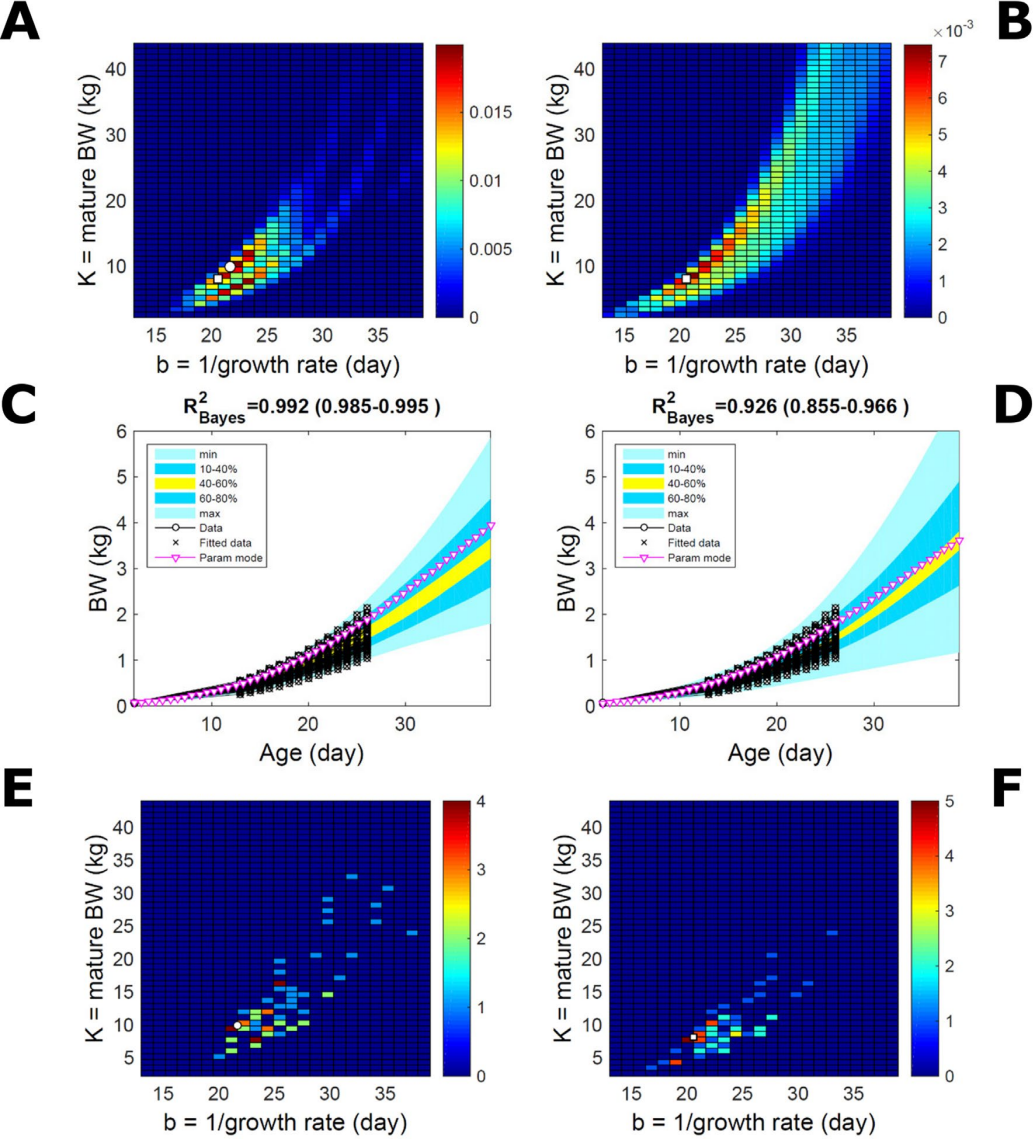
Body weight in a mixed-line chicken population. Estimation *via* ABC (left) and multiplicative-normal likelihood (right). **(A, B)** Population parameters posterior distribution (PD) of the BW Gompertz model. **(C, D)** Population predictive PD of BW. **(E, F)** scatterplot of individual mode parameters (K,b); colour scale indicates the number of individuals in each parameter cell. Data set with 71 individuals and even split between two genetic lines. Other details as in **Figures 2** and **7**.

This data set comprises an even split between two genetic lines, faster- and slower-growing; in the analysis we do not track which line each individual belongs to. Applying to this data set the approach applied to the pig population led to a parameter PD that is distinctively bimodal when obtained *via* ABC but has a single (faster-growing phenotype) mode when obtained *via* multiplicative-normal (**Figures 10A, B**) or additive-normal BL. Here, we interpret mode as a region of locally-high probability rather than as a point and, to visualise this detail, allow the probability density colour scale to differ between **Figure 10A** and **Figure 10B**. The BL parameter distribution (**Figure 10B**) identifies one phenotype mode in its fast-growing region but is relatively dispersed in the region of smaller-animal phenotypes (i.e., that grow less within the same timescale b). The ABC approach identifies the fast- and slow-growing lines with similar probability; this means that the BL approach does not fit well the slower-growing individuals. This difference between approaches is reflected in the population BW predictive PDs (**Figures 10C, D**): the ABC’s quantile range of 40–60% (yellow) correctly shifts upward and is wider than that of BL. Moreover, the BL’s goodness of individual fit (R^2^ > 0.85) is lower than the ABC’s (R^2^ > 0.98; c.f. **Figure S7** for individual values). The scatterplot of individual phenotype modes (**Figures 10E, F**) confirms the above findings. These results further suggest that ABC infers individual-specific trait distributions.

#### Body Weight in a Pig Population: Fitting Pooled Data

**Figure 11:**
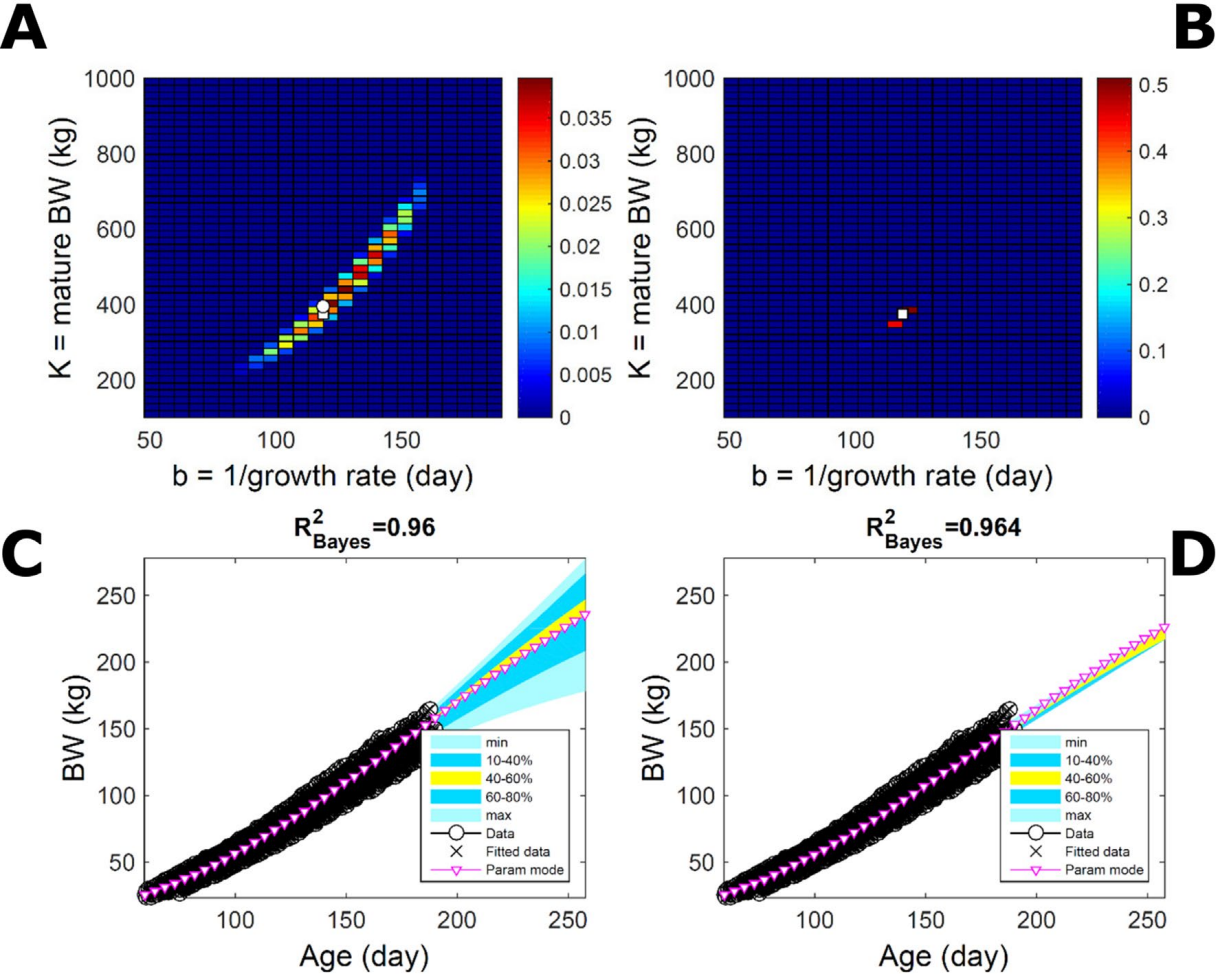
Body weight in a pig population: fitting pooled data. Estimation from the same data as in **Figure 7** using ABC (left) and multiplicative-normal likelihood (right), but fitting a single Gompertz curve to data pooled across individuals (that is, point observations from all individuals in the population fitted jointly). This approach is similar to statistical regression without individual-specific random effects and with nonparametric and normal error terms for the ABC and likelihood approaches, respectively. **(A, B)** Population parameter posterior distribution (PD) of the BW Gompertz model. **(C, D)** Population predictive PD of BW. Other details as in **Figure 9**.

To assess the approach to estimate population trait distributions based on fitting individuals separately (Equation 7), we compare with estimates based on fitting pooled data. We consider the case where data from multiple individuals are pooled as point observations (but not averaged over) and fitted jointly by an individual-trait model. This model represents an average individual in some sense but only in special cases in a statistical sense. Fitting the Gompertz curve (Equation 21) to pooled BW of the 50 pigs in **Figure 9**, the ABC approach leads to a population parameter PD (**Figure 11A**; **Table S1**) that shifts towards larger mature-size and timescale parameter values in relation to **Figure 9A**. The multiplicative-normal BL approach yields a parameter distribution with a similar mode but coalesces to nearly a point (**Figure 11B**; **Table S1**). The ABC’s population BW predictive-distribution (**Figure 11C**) is similar but with a more convex shape (later inflection point) than in **Figure 9C**, and beyond the data range has higher median and mode BW curves than in **Figure 9C**; the BL’s BW distribution has a similar median but is much narrower (**Figure 11D** versus **Figure 9D**). This pig population contains substantial trait variation among individuals (**Figures 9E, F**), whether caused by actual phenotypic differences or to distinct disturbances in the data; in this group, many animals have relatively lower growth that explains the lower population modes estimated *via* ABC or BL (**Figure 9**; **Table S1**). The shift in population distributions and increase in parameter mode and median BW, when fitting pooled data (**Figure 11**), are likely. The result of a greater influence from larger animals than when the population distributions build on individually-fitted distributions (Equation 7). When fitting pooled data, the ABC approach allowed estimation of uncertainty, whereas the normal BL approach was limited to central estimates of parameters and traits. Using additive-normal BL inference, the parameter distribution was similar but had a lower mode (**Table S1**) that influenced BW predictive-distribution (figure not shown).

## Discussion

We proposed a Bayesian modelling framework to infer individual- and population-level phenotypic variations from multiple-individual, multiple-observation trait data. The approach differs from established approaches by combining two steps: 1) *The use of nonparametric Bayesian inference (ABC) as a tool for fitting trait models to individual data* (Hypothesis 1, **Table 1**). The aim of this step is to derive data-based trait distributions that do not rely on an assumed residual distribution but rather on a data-driven form of noise; this distribution is often unknown [e.g., Barnard in discussion in Box (1980)] but must be assumed or guessed in parametric (likelihood-based) Bayesian approaches. 2) *Scaling-up from individual trait distributions to population phenotype distributions* (Hypothesis 2, Equation 7, **Table 1**). One aim of this step is to input maximal individual information (from stand-alone individual fitting in step 1) in a postinference population estimation. Another aim is to weigh individual contributions on probability rather than on trait value and to tackle outliers fairly (i.e., without having a disproportional influence). The focus here was on groups or populations within which trait variation results from individual responses (‘random effects’) to equal factors (‘fixed effects’, see Equation 7). The subsequent steps in the approach to compare phenotypes among groups or populations by estimating each group’s fixed effects on the traits are discussed later.

The relevance to animal or plant breeding, in particular, is two-fold: first, the approach’s potential for more accurate trait estimation could lead to a more accurate selection of individuals within a generation. Selection could be improved particularly where the estimation of breeding value involves multiple traits, given the approach’s potential to infer correlations accurately without requiring specification of a variance-covariance matrix. Second, the effect of a more accurate selection would build over generations and potentially lead to more decisive estimates of trait heritability because the selected groups would be less likely to mix individuals with disparate responses. Semiparametric approaches have been proposed with the same aim of Hypothesis 1 of weakening prior assumptions and shown to be potentially more accurate than fully parametric regression (Gonzalez-Recio et al., 2008). Other approaches [e.g., Drovandi et al. (2016)] have applied nonparametric Bayesian inference (as in hypothesis 1) to infer individual trait variations in a population but without the stand-alone individual fitting and the scaling-up of hypothesis 2. On the other hand, ABC is usually applied to complex or high-dimensional systems as a substitute for difficult-to-calculate likelihoods (Fearnhead and Prangle, 2012; Sunnaker et al., 2013) rather than as a general nonparametric tool (Green et al., 2015). Like hypothesis 2, other frameworks, Bayesian and frequentist such as DHGLM (Lee and Nelder, 2006), aim to capture and differentiate variation within and among individuals in a population but use parametric assumptions about within- and between-individual trait variations (Hill and Mulder, 2010; Cleasby et al., 2015). Multiple studies of individual trait variation have focused on ontogenetic growth using normal-likelihood methods (Strathe et al., 2010; Herault et al., 2011; Vincenzi et al., 2014) or non-normal inference (Zhang, 2016). The approach we propose is exploratory but could be alternative in suitable cases; it applies to individual trait models of general nonlinearity and complexity and tackles parameter correlations (Babtie and Stumpf, 2017). We demonstrated six advantages of the approach on parameter-parsimonious growth models that are widely used for the interpretability of their parameters as model-derived traits.

### Advantages of the Approach in Relation to Hypothesis 1

Application of the approach to single-individual ontogenetic growth supported Hypothesis 1 (**Table 1**). ABC inference was at least as accurate and as consistent as BL inference (Q1.1) in estimating growth and size traits from simulated data (**Figures 5**, **6**, and **8**), where distribution of noise is known, and from empirical data (**Figures 2**–**4**, **7** and **9**). In addition (Q1.2), ABC inferences (including out-of-data prediction) were more accurate than BL inferences when the likelihood distributional assumptions were not accurate or when the size of the data set (data volume) was reduced. The results on Q1.1 give confidence in the ABC approach, whereas those on Q1.2 demonstrate flexibility and potential robustness. In general, both the ABC and BL inferences were more accurate than those based on maximum likelihood estimation. In the case of simulated data, the findings were derived based on proximity to known targets. In the case of empirical data, support for ABC inference followed from its agreement with BL inference under one of the likelihood’s assumptions, additive or multiplicative noise. Both assumptions have been used (Blasco et al., 2003; Strathe et al., 2010; Coyne et al., 2017) and are plausible but yielded distinct inferences and could not be selected based on goodness of fit as measured by a Bayesian R^2^. There are therefore several advantages that arise from the methodology developed:

#### Automatic Form of the Noise Distribution

ABC does not require prior knowledge of the residual distribution of the trait data and thus does not require specification of a noise distribution to generate accurate inferences. BL inference, being sensitive to the parametric assumptions on this distribution is less reliable when there is limited or no knowledge about data distribution, which was clearly the case of the current empirical data. Even when such knowledge exists, there is an additional requirement to estimate auxiliary multitrait variance-covariance parameters (Ch. 8–9 in Blasco, 2017).

#### Robustness to Small Sample Size

ABC had greater robustness compared to likelihood inference in estimating modal and median growth curves when the volume of data per individual was low; this suggests that ABC requires fewer observations, and thus smaller experimental or monitoring resources, to achieve meaningful point estimates. Estimation of uncertainty, on the other hand, is affected by data volume in both approaches (Green et al., 2015). However, ABC gave accurate point estimates and did not require a large amount of data to stabilise its uncertainty estimates (c.f. area of higher probability among **Figures 4A**, **2A**, and **9A**, where data volume increased from 16 to 110 (×7) and to 4,900 (×45).

#### No Use of Unreliable Point (Mode-Based) Estimates

Both Bayesian approaches, ABC and BL, generated median predictive curves that were generally robust to challenges and more consistent between approaches than maximum likelihood predictive curves. MLE estimates were particularly sensitive to distributional assumptions and data volume and, in some cases, led to out-of-data curve projections with limited data support (i.e., far-from-median low-posterior-probability curves associated with parameter skew). It is well known that MLE estimates have restricted meaning in systems with poorly constrained parameters (Gutenkunst et al., 2007). Such point estimation issues are generally addressed through estimation of confidence intervals; however, the above caveats still apply when the accuracy of MLE and CIs relies on distributional assumptions.

### Advantages of the Approach in Relation to Hypothesis 2

Applying the approach to ontogenetic growth of multiple individuals in animal populations supported Hypothesis 2 (**Table 1**). The population trait and parameter PDs obtained *via* ABC or BL were similar and seemed plausible (Q2.1) when fitting empirical data from a population of a single genetic line (**Figures 9A–D**). However, there was a difference between approaches in the phenotypic pattern inferred across individuals (**Figures 9E, F**). The ABC-based phenotypic landscape had more outlier individuals than the BL-based landscape. The outliers represent either actual phenotypic differences or model misfit to perturbed growth trajectories; in the ABC approach, there was slightly greater and less variable goodness-of-fit across individuals (**Figure S6**). The BL-based phenotypic landscape, in turn, differed from a more-clustered pattern inferred *via* a second likelihood assumption. This distributional sensitivity and a lesser consistency in goodness-of-fit of the BL approach occurred despite there being parametric flexibility in the normal likelihood used to fit each individual (through joint estimation of variance parameters integrated out in the trait parameter marginal PDs, see Methods). These results support the likely possibility that trait distributions are individual-specific, and thus that the ABC fit may have been more accurate for some individuals (Q1.2), leading to the following consequences:

#### Identification of Individual Differences

An emerging strength of ABC is that it has the potential to more accurately identify trait differences among individuals, reflecting differences in genotype or in environmental factors, and thus to estimate trait variation within populations. ABC can flexibly capture the variation within each individual’s data as it does not rely on the residuals taking or deviating from a given form. Support for this adaptability was reinforced by fitting growth in a mixed-line bird population without inputting individual line information (**Figure 10**); ABC identified two clear phenotypic clusters, whereas the BL approach identified only one cluster (**Figures 10A, B, E, and F**). This outcome further illustrates that although hyperparameters (c.f. above) can capture some individual specificity in likelihood-based trait distributions, such approach is still limited by the sharing of features subsumed in parametric frameworks (Hill and Mulder, 2010; Cleasby et al., 2015). The potential of ABC for more accurate estimation of individual traits suggests it can facilitate the estimation of individual phenotypic variation (random effects) and even an automatic detection of some group effects (fixed effects) within a population (**Figure 10**). To characterise the pattern of individual phenotypes in a population, we used scatterplots of individual trait modes (**Figure 9** and **Figures 10E, D**); given the potential lack of robustness of mode estimates, alternative point estimates could be used to represent individuality in a population.

#### Tackling Outlier Individuals

A further advantage of the current framework relates to the tackling of outliers. At individual level, extreme variation can be addressed through the use of nonparametric approaches that allow for long-tailed residual distributions (Maier et al., 2017; Gianola et al., 2018). At the population level, the fact that the ABC and BL approaches inferred similar population phenotypic distributions (**Figure 9**) despite identifying different outlier individuals suggests that the population distribution (Equation 7) is robust to the presence of outliers (Q2.1). This property results from pooling individual contributions based on probability rather than on trait value, causing extreme-phenotype individuals to have an equal influence to other individuals and thus precluding trait-magnitude bias on population inferences and a need to remove outliers. In contrast, when a trait model is fitted not in stand-alone form but jointly to multiple individual data or to pooled data, as in many regression approaches, the interpretation of a population phenotypic distribution as a measure of variation in individual traits may be affected. When fitting jointly multiple individual data, there is some allowance for individual estimates to be influenced by other individuals (Cleasby et al., 2015). A more extreme case is the use of pooled, or even averaged, data, which in general does not follow an individual pattern [e.g., consider pooling live weight by age of individuals with differing birth weight or delay in growth (Begall, 1997)]. Redoing the fitting of **Figure 9** but on pooled data (point observations without individual tags), there was still characterisation of phenotypic variation in the ABC-based population distributions, but convergence to a central estimate in the BL-based inference as the increased volume of fitted data leads to very narrow PDs (**Figure 11**). The population trait distributions derived from pooled data, rather than from individually fitted distributions, had a shift towards larger trait values, likely because here the influence of each individual’s data is determined by the individual’s trait magnitude (Q2.2). Other studies have also found improvement in prediction by fitting individual rather than population data (e.g., Vincenzi et al., 2014).

#### Population Characteristics

A final advantage of the current framework is that, by fitting individuals in stand-alone form, it is possible to infer more accurate population distributions, as well as central estimates; it is also possible to track individual phenotype patterns within a population (as in **Figures 9**, **10E, F**). An improved estimation of population distributions could also offer a better characterisation of phenotypic differences among populations.

### Extension and Limitations of the Approach

#### Indices of Intraindividual Variation

Phenotype comparisons among studies, populations, or groups can be made, for example, using the average and range of variation in the population posterior trait distributions (**Figures 9**, **10A–D**) or scatterplots of individual point estimates (**Figures 9**, **10E, F**). However, we can separate intraindividual from interindividual variation using population indices of individual variation. For example, repeatability in behaviour ecology (Cleasby et al., 2015) measures variation in the individual predictability of a trait, a measure of plasticity. A variation index (ρ) can be defined in the current nonparametric Bayesian context. For example, evaluating a summary statistic (ρ_i_) of the trait PD of individual i, such as a normalised sample variance or a credible interval, then ρ would be a statistic on the population distribution of ρ_i_, such as the variance of the raw distribution or of a fitted parametric distribution. These extensions could be studied in future work. Given that these indices are derived postinference, Bayesian inference is not influenced by their choice.

#### Fixed and Random Effects

The approach proposed in this paper can be extended to estimate the influence of population-level (fixed-effect) factors on modelled traits, in addition to estimating individual-level (random effect) factors, which has been the focus so far. In the population examples studied (**Figures 9**–**11**), it was assumed that all individuals were subject to equal population or group conditions (e.g., a line, diet, or macroenvironment, corresponding to fixed population parameters θ_P_ and covariates in the trait model, section *A Probabilistic Framework for Modelling Phenotypes in Individuals and Populations*) and that the individuals responded independently to such conditions. This variation in responses (represented in the trait model as variation in the parameters θ_i_, section *A Probabilistic Framework for Modelling Phenotypes in Individuals and Populations*) could have resulted from individual factors, for example, genetic or acquired differences, or from nonmeasurable microenvironment variation (Hill and Mulder, 2010). When both the responses and the factors affecting them differ among the individuals, the conditional independence of the responses does not strictly apply (section *A Probabilistic Framework for Modelling Phenotypes in Individuals and Populations*). The responses are correlated through the extent to which individuals share similar conditions, which can confound the estimates of the responses. The two-line chicken population (**Figure 10**) was a deliberate exception to the assumption. Here, a clear distinction emerged between the parameters of the two subgroups (when using ABC) because the line effect was predominantly binary and enough to override confounding. For a generic population where the individuals are subject to distinct factors (represented by distinct values of the parameters θ_P_ and covariates X in the trait model), we follow the assumption that hierarchical mixed-effect regression models make (Hill, 2010; Gelman et al., 2013; Blasco, 2017) to distinguish random from fixed effects, that is, that individuals can be categorised into equal-factor groups according to the values of θ_P_ and X. The remaining question is how to estimate all the parameters of the model, θ_i_ and θ_P_. One possible method is to apply the approach of section *A Probabilistic Framework for Modelling Phenotypes in Individuals and Populations* separately to each group, to calculate group-specific posterior averages of the modelled responses or of the parameters of the model, and then to compare the group-specific phenotype averages. The fixed-effects and interactions are given by the differences between phenotype averages of suitable groups. This method builds on the notion of fixed-effects as group-specific additive components of the responses (Blasco, 2017) or of the parameters (Coyne et al., 2017), and is computationally simple as it is postinferential to the Bayesian approach in this paper. In the case where the model is a linear additive mixed-effect model, it is easy to relate fixed effects on responses and on parameters; here, the outcomes of this method are expected to be equivalent to those of mixed-effect regression because the mean of the random effects is zero. In the case of nonlinear mixed-effect models, the estimates obtained *via* either method may or may not be similar. Further formalisation of this approach could be further work. A second method would be to estimate jointly the two types of effects. The iterative estimation would relate to iterative approaches used in many hierarchical regression models (Coyne et al., 2017) but would be Bayesian. As a joint estimation of individual- and group-level parameters would depend directly on the number of individuals, it could become an inferential problem of high dimension (in parameters and data). One approach to the problem is to fit trait models jointly to multiple individuals (e.g., Cleasby et al., 2015). Alternatively, Bayesian parameter sampling schemes mixing group-parameter proposals and stand-alone fitting of individual data could be feasible, depending on balancing numbers of parameters and individuals and computation. There has been progress in developing ABC techniques for sampling correlated parameters in such high-dimensional problems (e.g., Kousathanas et al., 2016).

#### Interpretation of ABC

There is growing interest in the application of ABC for its flexibility in tackling large complex inferential problems (Robert et al., 2011; Fearnhead and Prangle, 2012; Sunnaker et al., 2013), but there are also limitations and criticisms to ABC, most referring to those systems. First, when used as an approximation to a problem with a known data distribution, ABC produces a sample from a PD that is not the exact one supported by the likelihood as the tolerance parameter is non-zero (Robert et al., 2011; Wilkinson, 2013; Kypraios et al., 2017). However, this point is less relevant when the data distribution is not known with confidence, which is the case we consider. Moreover, in the simulated-data examples, we did not find evidence of poor approximation. Second, prior parameter assumptions can influence inference. We took a least-assumption stand by adopting wide uniform prior distributions (suggested directly by the data range, and whose potential computational cost was tackled through methods in Text S1), and diagnosed the posterior distributions. Third, ABC may not reliably discriminate competing trait models when fitting insufficient summary statistics as loss of information can cause misidentification (Robert et al., 2011). The data and models in the case studies, while realistic and having shown worth of applying this nonparametric approach, were simple enough not to demand the use of such statistics. Moreover, we tested alternative noise models but not competing trait models as that was not the purpose here; however, comparing alternative models for ontogenetic growth based on the procedures presented here is desirable and feasible [e.g. as in Toni et al. (2009)]. Finally, the level of computation required by ABC depends on the problem’s dimensions, that is, number of traits, individuals, and model parameters. We showed examples with up to four model parameters, but higher-dimensional applications are emerging, for example, more than 10 parameters in systems biology (van der Vaart et al., 2015; Drovandi et al., 2016; Saa and Nielsen, 2016; Daly et al., 2017), although not necessarily using multiple-individual data.

## Conclusions

We proposed a nonparametric approach to quantify phenotype variations in groups and populations of organisms. This work may be relevant to areas where phenotype variation is important and, more generally, to areas where there is interest in methods to fit mathematical models to biological data with unknown data distribution, with outliers, and with few or irregular observations per individual.

## Ethics Statement

The empirical data used in this paper were not generated in this study. The data originated from animals treated under normal husbandry procedures and for this reason no Institutional or other relevant ethics board approval was required for its collection.

## Author Contributions

JF conceptualised, developed, and tested the methods; analysed the data; and wrote the original draft. JF and IK designed the study, acquired data, verified the results, and reviewed and edited the manuscript. IK acquired the funding. Both authors approved the final version of the manuscript for publication.

## Funding

This research was funded in part by the European Commission (Grant agreement no: 633531) under the EU Framework Programme for Research and Innovation Horizon 2020. The Commission accepts no responsibility or liability whatsoever with regard to the material in this paper. The funders had no role in study design, data collection and analysis, decision to publish, or preparation of the manuscript.

## Conflict of Interest Statement

The authors declare that the research was conducted in the absence of any commercial or financial relationships that could be construed as a potential conflict of interest.

## Abbreviations

ABC, approximate Bayesian computation; BL, Bayesian likelihood; BW, body weight; DHGLM, double hierarchical generalised linear models; CrI, credible interval; MCMC, Markov chain Monte Carlo; MLE, maximum likelihood estimation (or estimator); MVN, multivariate normal (probability distribution); NEI, net energy intake; PD, posterior distribution (i.e., parameter PD or trait-predictive PD).

## Supplementary Material

Text S1 Algorithm for Approximate Bayesian Computation Markov-chain Monte Carlo.

Table S1 Results and assessment of fitting the growth model to the datasets.

Figure S1 Body weight of individual pig: multiplicative-normal likelihood.

Figure S2 Simulated normally-distributed individual trait: residuals.

Figure S3 Simulated skew-distributed individual trait: diagnostic of ABC-MCMC Convergence.

Figure S4 Body weight and energy intake of individual pig: fitting the full dataset.

Figure S5 Body weight in a pig population: additive-normal likelihood.

Figure S6 Body weight in a pig population: Goodness of fit across individuals.

Figure S7 Body weight in a genetically-heterogeneous chicken population: Goodness of fit across individuals.
