# Supplementary material for: Bayesian, Likelihood-Free Modelling of Phenotypic Plasticity and Variability in Individuals and Populations
Source: Front Genet. 2019 Sep 20;10:727. doi: 10.3389/fgene.2019.00727 (PMC6764410; doi:10.3389/fgene.2019.00727)
Supplement: Text S1 — Algorithm for Approximate Bayesian Computation Markov-chain Monte Carlo. [file Presentation_1.pdf]

Supplementary Material

Bayesian, likelihood-free modelling of phenotypic plasticity and variability in individuals and populations

Joao A.N. Filipe* **and Ilias Kyriazakis**

Agriculture, School of Natural and Environmental Sciences, Newcastle University, Newcastle upon Tyne, United Kingdom.

*** Correspondence:** Corresponding Author: joao.filipe@newcastle.ac.uk

Text S1 - Algorithm for Approximate Bayesian Computation Markov-chain Monte Carlo

The algorithm used for sampling the model parameter space and to estimate an approximate posterior parameter distribution, incorporates simulation of the model traits M (Equations 1, 6, 21) into a standard Metropolis-Hastings (M-H) Markov Chain Monte Carlo (MCMC) algorithm (Gilks et al 1997). The ABC-MCMC algorithm (Marjoram et al. 2003) was designed to overcome issues associated with low acceptance probability, which occur when sampling directly from a prior distribution that differs substantially from the target parameter distribution. The algorithm has the following structure (c.f. Toni et al 2009, Kypraios et al 2016).

ABC-MCMC algorithm

Input: observed temporal data $D(t_D)$, where t_D is a vector of time points, summary statistics $s(\cdot)$, distance function d(.,.), tolerance $\mathcal{E}$, proposal distribution $q(\cdot|\cdot)$, prior distribution $\pi(\theta)$, initial state of Markov chain θ^0.

Output: samples from $\tilde{\pi}(.|D) = \pi(.|D, d(s(D), s(.)) < \varepsilon)$, the approximation to the posterior distribution $\pi(.|D)$.

1: Initialise the step number and state of the Markov chain: c=1 and $\theta^c = \theta^0$.

2: Sample θ^* from a proposal distribution $q(\cdot|\theta_c)$.

3: Simulate a dataset D^* from the continuous-time model $M(t,\theta)$ at $t=t_D$ and $\theta=\theta^*$.

4: Accept θ* with probability:

(S.1) $\alpha = \min\left(1, \frac{r(D^*,\theta^*)\,\pi(\theta^*)\,q(\theta^*|\theta_c)}{r(D^c,\theta_c)\,\pi(\theta_c)\,q(\theta_c|\theta^*)}\right)$

(S.2) $r(D^*, \theta^*) = \mathbb{I}(d(s(D), s(D^*)) < \varepsilon)$,

where $\mathbb{I}(x)$=1 if x is true and 0 otherwise.

5: if θ^* is accepted, set the new state $\theta_{c+1} = \theta^*$, otherwise $\theta_{c+1} = \theta_c$.

6: Set c=c+1; go to 2 and repeat until the required sample size is reached.

For a uniform prior distribution and a symmetric proposal distribution, the probability of acceptance is 1 if $d(s(D), s(D^*)) < \varepsilon)$ and 0 otherwise. The following choices were made (see M&M): distance in Eq (12), tolerance start value $\mathcal{E}_{est}$ based on the data according to Eq (13) and tuned for each trait fitted if the corresponding acceptance rate is zero; a multivariate-normal proposal distribution centred at the current state with variance-covariance estimate based on the chain up to the current state (approach similar to that in Rosenthal (2010)), and burn-in period of one third of the chosen chain length. While, the chain generated by ABC-MCMC is guaranteed to converge asymptotically to a stationary posterior distribution (Majoran et al 2003), in practice, it is possible the chain may take too long to converge to be computationally viable, depending on the start parameter point and the nature and dimension of the model and data. To tackle unsuitable start points in regions with negligible parameter support, i.e. zero proposal acceptance within the chain length, we used sequential sampling of start points across sectors in parameter space up to a maximum number of trials. More efficient algorithms that attempt to overcome these difficulties and tune the choice of tolerances, such as sequential Monte Carlo ABC (Sisson et al 2007, Toni et al 2009, Del Moral 2012), have been developed, using multiple simultaneous chains (particles) with different start values that are filtered through using suitable probability weights. For the low-dimensional examples used in this paper it is sufficient to use ABC-MCMC, whose convergence is shown to be attained in moderate-length chains. For the likelihood-based inference, an identical M-H MCMC algorithm applies, with the difference that the r ratio in the probability of acceptance ((S.2) and (S.1)) is replaced by a likelihood ratio. In this paper, however, a direct parameter-grid calculation is used for the likelihood-based parameter posterior distribution.

Del Moral, P., A. Doucet, and A. Jasra. 2012. An adaptive sequential Monte Carlo method for approximate Bayesian computation. Stat. Comput. 22: 1009-1020.

Gelman, A. B. et al. 2013. Bayesian Data Analysis, Third Edition, 3rd Edition. CRC Press, [S.l.].

Gilks, W. R., S. Richardson, and D. J. Spiegelhalter. 1997. Markov chain Monte Carlo in practice. Chapman and Hall, London.

Marjoram, P., J. Molitor, V. Plagnol, and S. Tavare. 2003. Markov chain Monte Carlo without likelihoods. Proceedings of the National Academy of Sciences of the United States of America 100: 15324-15328.

Rosenthal, J. R. 2010. Optimal Proposal Distributions and Adaptive MCMC. In: S. Brooks, A. B. Gelman, G. L. Jones and X.-L. Meng (eds.) Handbook of Markov Chain Monte Carlo. Chapman and Hall/CRC, Boca Raton.

Sisson, S. A., Y. Fan, and M. M. Tanaka. 2007. Sequential Monte Carlo without likelihoods. Proceedings of the National Academy of Sciences of the United States of America 104: 1760-1765.

Toni, T., D. Welch, N. Strelkowa, A. Ipsen, and M. P. H. Stumpf. 2009. Approximate Bayesian computation scheme for parameter inference and model selection in dynamical systems. J. R. Soc. Interface 6: 187-202.
