# Supplementary material for: Bayesian, Likelihood-Free Modelling of Phenotypic Plasticity and Variability in Individuals and Populations
Source: Front Genet. 2019 Sep 20;10:727. doi: 10.3389/fgene.2019.00727 (PMC6764410; doi:10.3389/fgene.2019.00727)
Supplement: Table S1 — Results and assessment of fitting the growth model to the datasets. [file Table_1.pdf]

1 – Individual dataset

Dataset	Section	Full data				Reduced data (6% or 25%)	
**Pig**	**3.1.1**	**ABC** (Fig 2)	**BL-an** (Fig 2)	**BL-mn** (Fig S1)		**ABC** (Fig 4)	**BL-an** (Fig 4)
	BW (K, b)	467kg, 118d	504kg, 124d<br>>ABC mode	376kg, 107d<br><ABC mode		467kg, 118d	559kg, 129d<br>>ABC mode
	R_{Bayes}^2	0.998	0.996	0.994		1.000	0.989
**simulated**	**Sec 3.1.2**	**ABC** (Fig 5)	**BL-an** (Fig 5)		**Target**		
	BW (K, b)	100, 50.3	100, 50.3		100, 50		
	R_{Bayes}^2	0.995	0.995				
**simulated**	**3.1.3**	**ABC** (Fig 6)	**BL-an** (Fig 6)	**BL-mn**			
	BW (K, b)	95.8, 50.3	91.7, 48.8	95.8, 51.9	100, 50		
	R_{Bayes}^2	0.974	0.970	0.963			
**Pig**	**3.2.1**	**ABC** (Fig S4)	**BL-an** (Fig S4)			**ABC** (Fig 7)	**BL-an** (Fig 7)
	BW (K, b)	400kg, 110d				400kg,110d	400kg, 110d
	R_{Bayes}^2	0.998	0.997			0.9997	0.999
	NEI (K2, b2)	45MJ/d, 65d	45MJ/d, 65d			45MJ/d, 65d	75MJ/d ,91d
	R_{Bayes}^2	0.793	0.784			0.850	0.856
**simulated**	**3.2.2 (a)**	**ABC** (Fig 8)	**BL-an** (Fig 8)		**Target**		
	BW (K, b)	100, 51	100, 51		100, 50		
	R_{Bayes}^2	0.991	0.965				
	NEI (K2, b2)	28, 43	24, 29		25, 30		
	R_{Bayes}^2	0.714	0.35				

Residual diagnostics of the predicted traits: The residuals from the ABC and BL approaches show no obvious temporal pattern or trend for each individual fit (Section 3.1.1-3, 3.2.1-2), particularly when scaled by size. Examples are given for the BW of a pig (Figure 3) and for a simulated normally-distributed trait (Figure S2).

ABC-MCMC Diagnostics: The MCMC sample showed good chain mixing and convergence in each individual fit (Sections 3.1.1-3, 3.2.1-2); this is shown for the simulated skew-distributed trait (Figure S3).

2 – All individual's datasets in a population

**Pig population**	**Sec 3.3.1**	**ABC** (Fig 9)	**BL-an** (Fig S5)	**BL-mn** (Fig 9)
	BW (K, b)	230kg, 78d	278kg, 83d	230kg, 78d
	$\mathbf{R_{adj}^2}$	0.988 – 0.996	0.977 – 0.996	0.975 – 0.994
**Pig population**	**Sec 3.3.3**	**ABC** (Fig 11)	**BL-an**	**BL-mn** (Fig 11)
	BW (K, b)	394kg, 119d	320kg, 107d	375kg, 119d
	$\mathbf{R_{adj}^2}$	0.96	0.966	0.964

Table S1. Results and assessment of fitting the growth model to the datasets. (1) Mode of PD of model parameter (K,b). (2) Goodness of fit of the trait predictive PD to the dataset (R_{Bayes}^2). (3) Residual diagnostics of the predicted traits. (4) ABC-MCMC Diagnostics. The parameter of the Gompert model (Equation 21) are K, mature size, and b, maturation time scale. Section refers to the 'Results'. BL-an=BL-additive-normal, BL-ml=BL-multiplicative-normal. (a) Variance-covariance $(\sigma_1^2\ \rho;\ \rho\ \sigma_2^2) = (2^2, 0.1; 0.1, 4^2)$; the noise variance (Figures 8E-H) is exaggerated in relation to Figure 7 to assess the potential of the inference to tackle noisy data. (b) Quantitatively different from individuals in Figures 2 and 7.
