# Supplementary material for: Bayesian, Likelihood-Free Modelling of Phenotypic Plasticity and Variability in Individuals and Populations
Source: Front Genet. 2019 Sep 20;10:727. doi: 10.3389/fgene.2019.00727 (PMC6764410; doi:10.3389/fgene.2019.00727)
Supplement: Figure S1 — Body weight of individual pig: multiplicative-normal likelihood. [file Image_1.pdf]

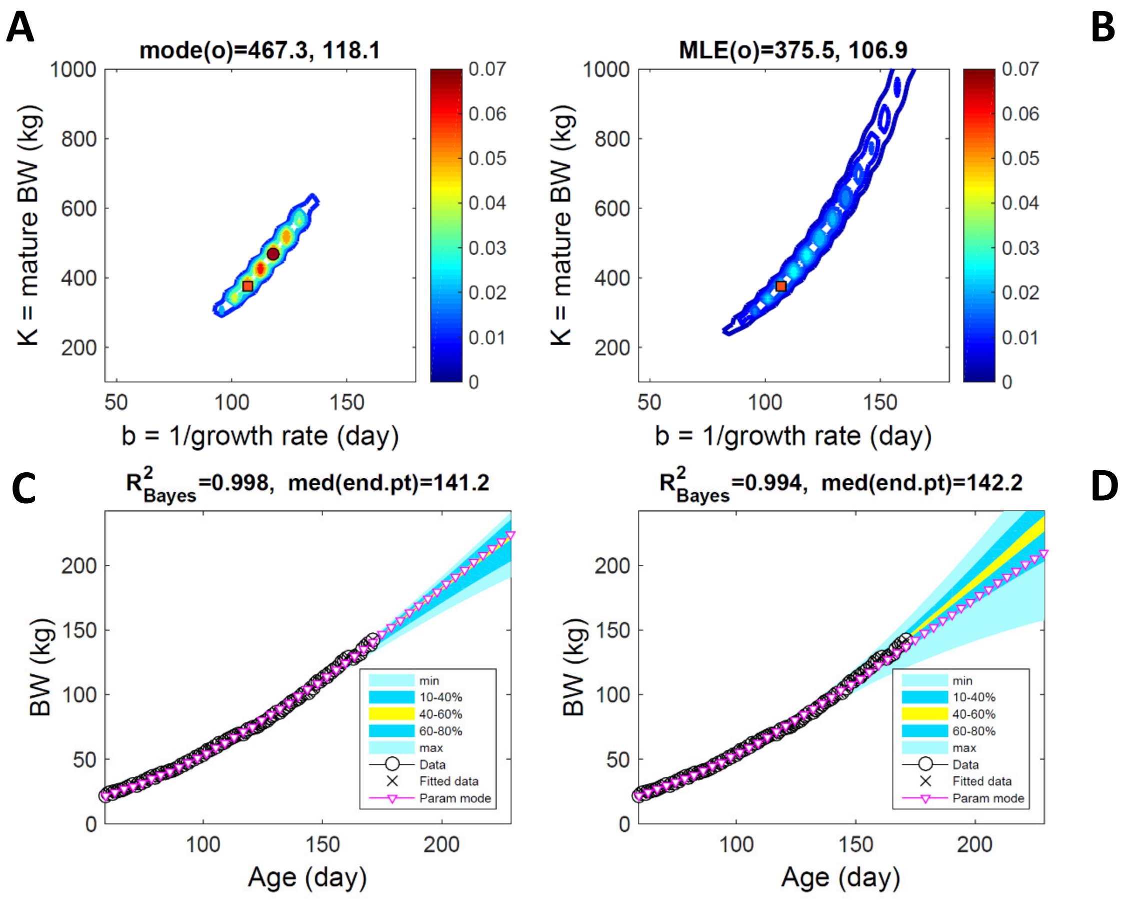

Figure S1 | Body weight of individual pig: multiplicative-normal likelihood. Trait parameters and temporal distribution estimated via ABC (left) and normal likelihood (right) as in Figure 2, but assuming a likelihood function for multiplicative-normal rather than additive-normal noise (Equation 15). **Top (A,B):** Parameter posterior distribution of the Gompertz model. **Bottom (C,D):** Predictive posterior distribution of body weight. Other detail as in Figure 2.
