# Supplementary material for: Bayesian, Likelihood-Free Modelling of Phenotypic Plasticity and Variability in Individuals and Populations
Source: Front Genet. 2019 Sep 20;10:727. doi: 10.3389/fgene.2019.00727 (PMC6764410; doi:10.3389/fgene.2019.00727)
Supplement: Figure S2 — Simulated normally-distributed individual trait: residuals. [file Image_2.pdf]

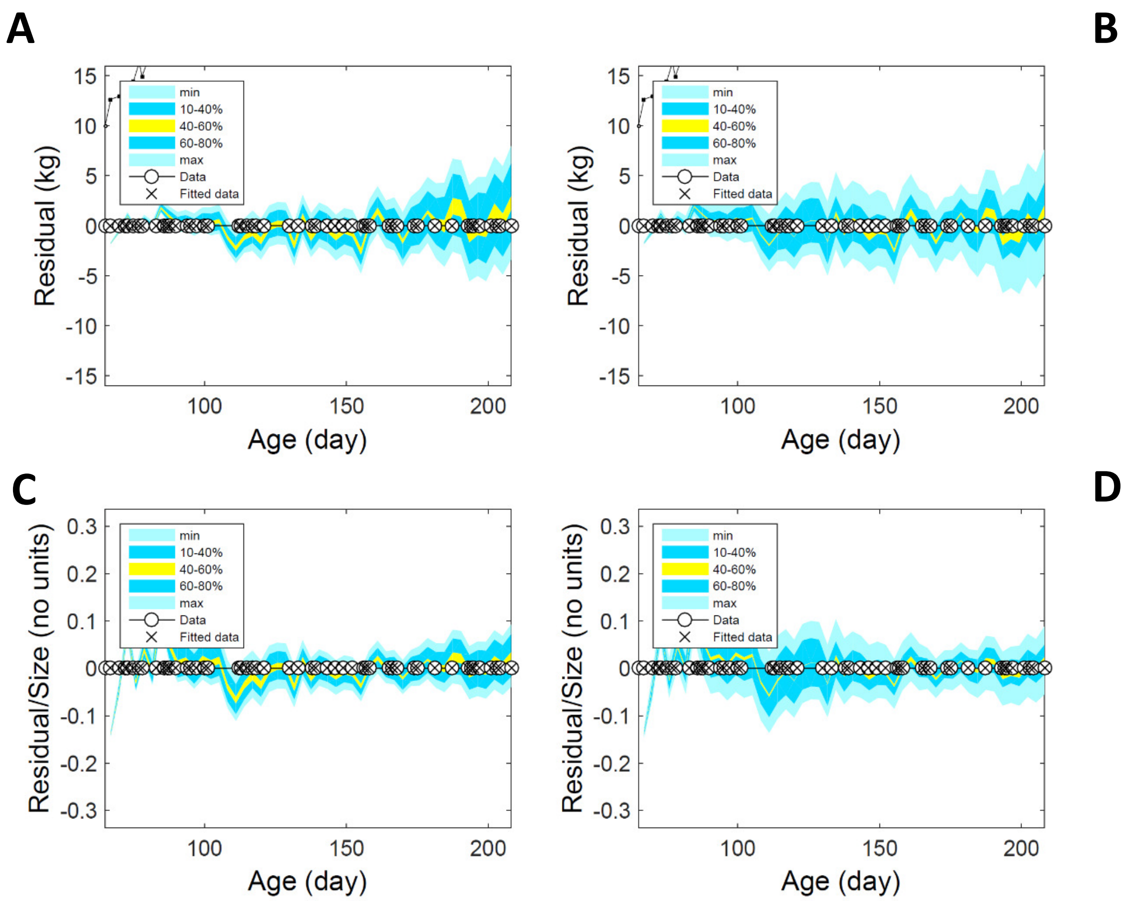

Figure S2 | Simulated normally-distributed individual trait: residuals. Residual difference between the fitted data (48 points) and growth model (Equation 21) fitted in Figure 5 via ABC (left) and via additive-normal likelihood (right). Colour shows cumulative probability within the quantile ranges in legend (median is the centre of the yellow range). The increased range of the residual distribution at the far end (larger age) is due to reduced model constraint in fitting at this end.
