# Supplementary material for: Bayesian, Likelihood-Free Modelling of Phenotypic Plasticity and Variability in Individuals and Populations
Source: Front Genet. 2019 Sep 20;10:727. doi: 10.3389/fgene.2019.00727 (PMC6764410; doi:10.3389/fgene.2019.00727)
Supplement: Figure S3 — Simulated skew-distributed individual trait: diagnostic of ABC-MCMC Convergence. [file Image_3.pdf]

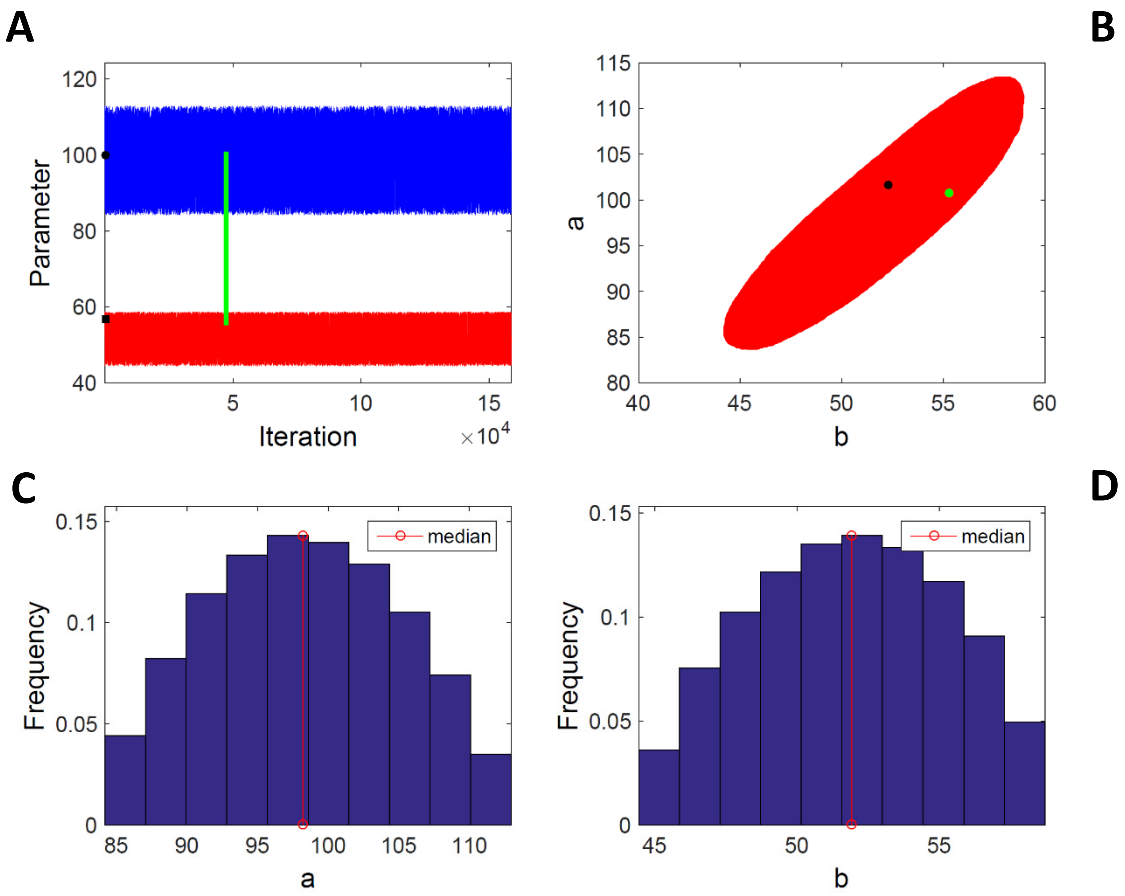

Figure S3 | Simulated skew-distributed individual trait: diagnostic of ABC-MCMC convergence. A) Sampled parameters K (blue) and b (red) versus iteration rank. **B)** Point distribution of the sample and parameter correlation. **C-D)** Marginal sample distributions of K and b, respectively. Green indicates position of burn-in; black is start of chain.
