# Supplementary material for: Bayesian, Likelihood-Free Modelling of Phenotypic Plasticity and Variability in Individuals and Populations
Source: Front Genet. 2019 Sep 20;10:727. doi: 10.3389/fgene.2019.00727 (PMC6764410; doi:10.3389/fgene.2019.00727)
Supplement: Figure S4 — Body weight and energy intake of individual pig: fitting the full dataset. [file Image_4.pdf]

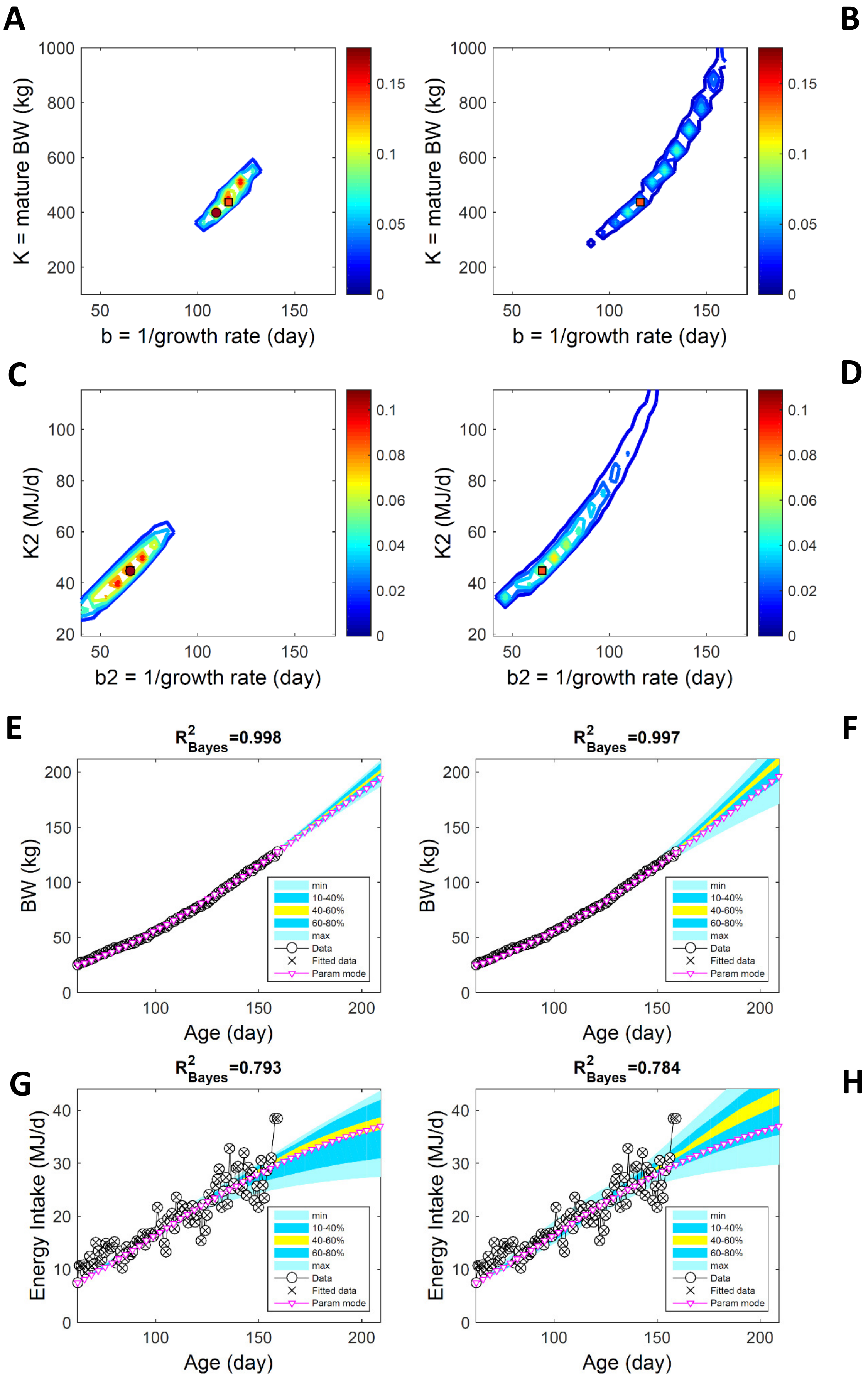

A
B
C
D
E
F
G
H
K = mature BW (kg)
b = 1/growth rate (day)
K2 (MJ/d)
b2 = 1/growth rate (day)
R2 Bayes =0.998
R2 Bayes =0.997
R2 Bayes =0.793
R2 Bayes =0.784
BW (kg)
Energy Intake (MJ/d)
Age (day)
min
10-40%
40-60%
60-80%
max
Data
Fitted data
Param mode

Figure S4 | Body weight and energy intake of individual pig: fitting the full dataset. Trait parameters and temporal distribution estimated from two observed correlated traits of the same individual using ABC (left) and additive-multivariate-normal likelihood (right) (Equation 15). The full dataset is fitted. Details as in Figure 7, where only 25% of the dataset is fitted.
