# Supplementary material for: Bayesian, Likelihood-Free Modelling of Phenotypic Plasticity and Variability in Individuals and Populations
Source: Front Genet. 2019 Sep 20;10:727. doi: 10.3389/fgene.2019.00727 (PMC6764410; doi:10.3389/fgene.2019.00727)
Supplement: Figure S5 — Body weight in a pig population: additive-normal likelihood. [file Image_5.pdf]

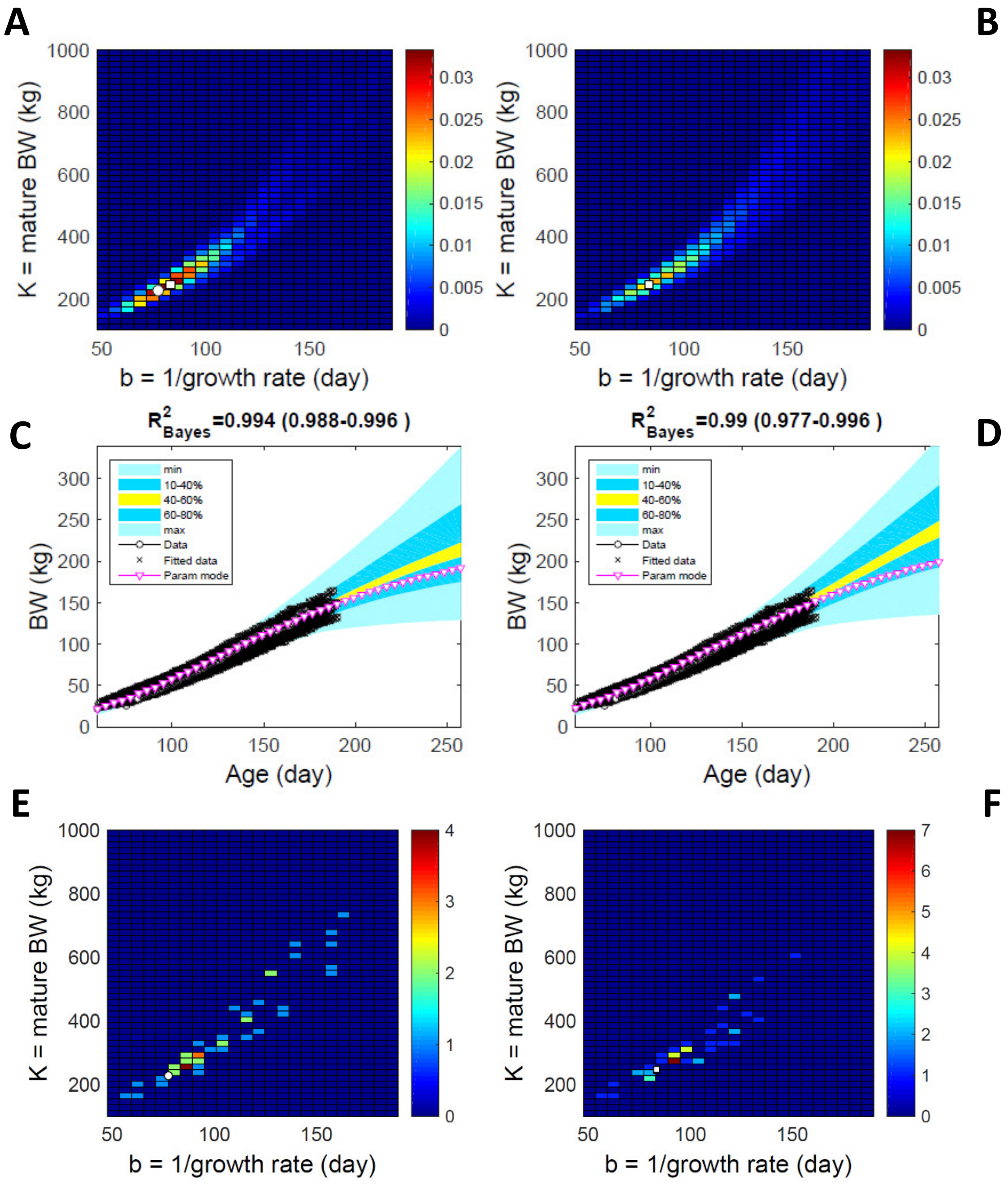

Figure S5 | Body weight in a pig population: additive-normal likelihood. Estimation via ABC (left) and additive-normal likelihood (right). **Row 1 (A,B):** Population parameter posterior distribution of the body weight Gompertz model. **Row 2 (C,D):** Population predictive posterior distribution of body weight. **Row 3 (E,F):** scatterplot of individual mode parameters (K,b); colour scale indicates number of individuals with parameter mode within each cell. Other detail as in Figures 2 and 9.
