# Supplementary material for: Bayesian, Likelihood-Free Modelling of Phenotypic Plasticity and Variability in Individuals and Populations
Source: Front Genet. 2019 Sep 20;10:727. doi: 10.3389/fgene.2019.00727 (PMC6764410; doi:10.3389/fgene.2019.00727)
Supplement: Figure S6 — Body weight in a pig population: Goodness of fit across individuals. [file Image_6.pdf]

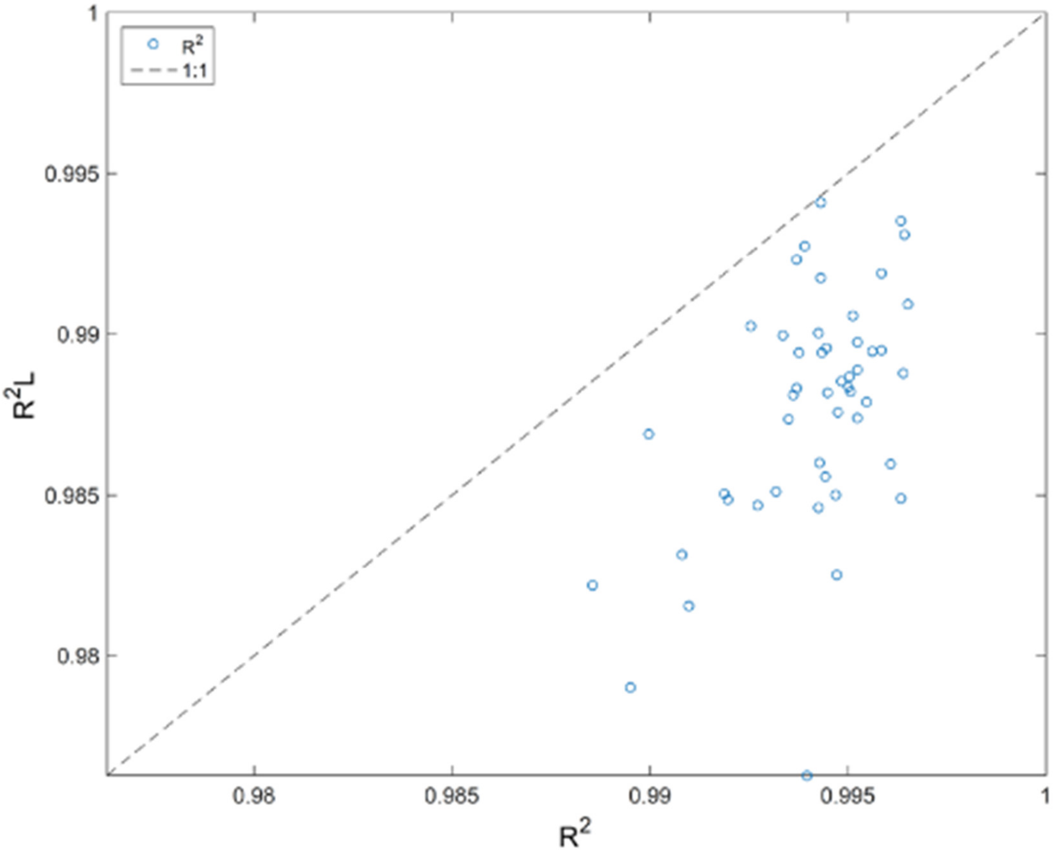


**Figure S6 | Body weight in a pig population: Goodness of fit across individuals.** Goodness of fit of the likelihood and likelihood-free approaches ($R_L^2$ and $R^2$) across individuals in the population
