# Supplementary material for: Bayesian, Likelihood-Free Modelling of Phenotypic Plasticity and Variability in Individuals and Populations
Source: Front Genet. 2019 Sep 20;10:727. doi: 10.3389/fgene.2019.00727 (PMC6764410; doi:10.3389/fgene.2019.00727)
Supplement: Figure S7 — Body weight in a genetically-heterogeneous chicken population: Goodness of fit across individuals. [file Image_7.pdf]

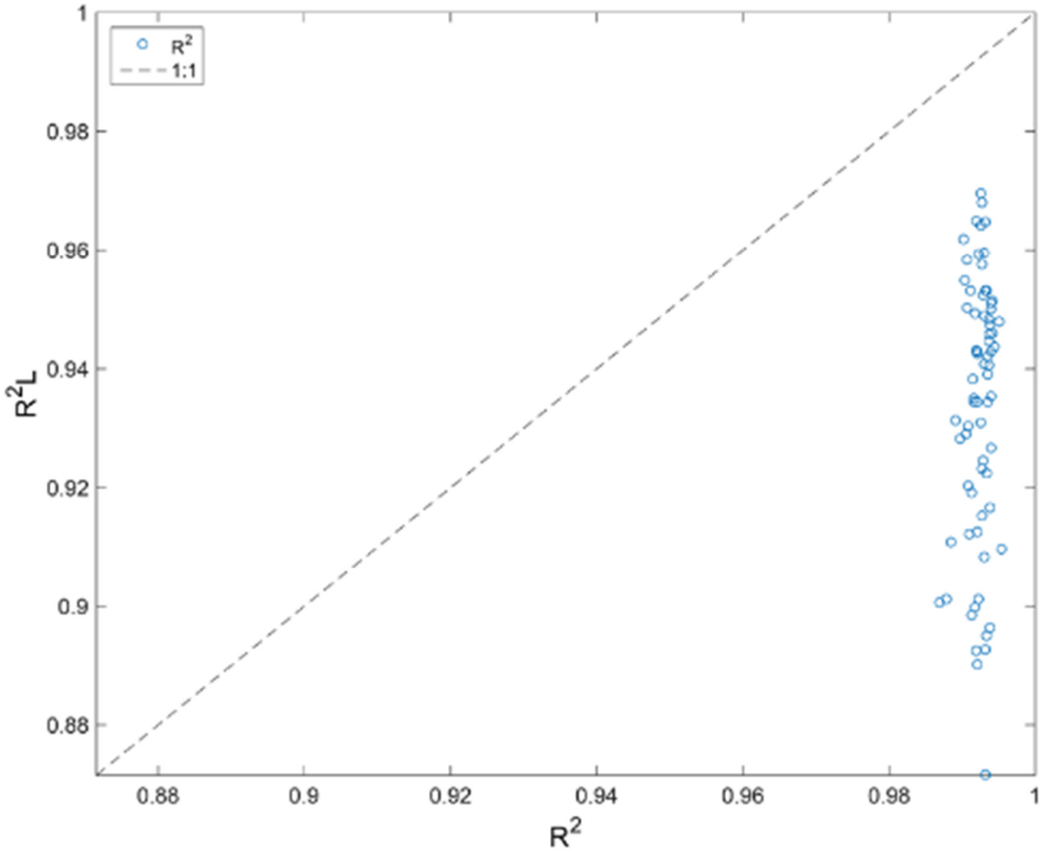

Figure S7 | Body weight in a genetically-heterogeneous chicken population: Goodness of fit across individuals. Goodness of fit of the likelihood and likelihood-free approaches (R_L^2 and R^2) across individuals in the population.
